# Biological effects in normal human fibroblasts following chronic and acute irradiation with both low- and high-LET radiation

**DOI:** 10.3389/fpubh.2024.1404748

**Published:** 2024-10-22

**Authors:** Pasqualino Anello, Giuseppe Esposito

**Affiliations:** ^1^Istituto Superiore di Sanità (ISS), Rome, Italy; ^2^Istituto Nazionale di Fisica Nucleare (INFN) Sezione Roma 1, Rome, Italy

**Keywords:** low-LET radiation, high-LET radiation, chronic exposure, acute exposure, human fibroblast, dose and dose rate effectiveness factor

## Abstract

**Introduction:**

Radiobiological studies at low dose rates allow us to improve our knowledge of the mechanisms by which radiation exerts its effects on biological systems following chronic exposures. Moreover, these studies can complement available epidemiological data on the biological effects of low doses and dose rates of ionizing radiation. Very few studies have simultaneously compared the biological effects of low- and high-LET radiations at the same dose rate for chronic irradiation.

**Methods:**

We compared, for the first time in the same experiment, the effects of chronic (dose rates as low as ~18 and 5 mGy/h) and acute irradiations on clonogenicity and micronucleus formation in AG1522 normal human skin fibroblasts in the confluent state exposed to doses of low- and high-LET radiation (gamma rays and alpha particles) to investigate any differences due to the different radiation quality and different dose rate (in the dose range 0.006–0.9 Gy for alpha particles and 0.4–2.3 Gy for gamma rays).

**Results:**

As expected, alpha particles were more effective than gamma rays at inducing cytogenetic damage and reduced clonogenic cell survival. For gamma rays, the cytogenetic damage and the reduction of clonogenic cell survival were greater when the dose was delivered acutely instead of chronically. Instead, for the alpha particles, at the same dose, we found equal cytogenetic damage and reduction of clonogenic cell survival for both chronic and acute exposure (except for the highest doses of 0.4 and 0.9 Gy, where cytogenetic damage is greater at a low dose rate).

**Conclusion:**

The results of this study may have an impact on space and terrestrial radioprotection of humans at low doses and low dose rates, on biodosimetry, and on the use of ionizing radiation in medicine. These results also provide insights into understanding damage induction and cell reaction mechanisms following chronic exposure (at dose rates as low as 18 and 5 mGy/h) to low- and high-LET radiation.

## 1 Introduction

It is well-known that ionizing radiation, interacting with the cells, induces different types of DNA damage, which underlie the biological effects of ionizing radiation. DNA double-strand breaks (DSBs) are generally considered the most biologically relevant DNA lesions produced by ionizing radiation (1–3).

In the last three decades, a continuously growing interest in the biological effects induced by low doses/low dose rates of ionizing radiation, which is relevant to environmental and occupational radiation exposure, has arisen in the scientific community (4, 5). For example, a significant problem is that of exposure to the naturally occurring radioactive gas radon and its resulting alpha particles emitting progeny that is now known to be a major cause of lung cancer (6). On the other hand, human exposure to low doses of ionizing radiation, both low and high linear energy transfer (LET), continuously increases. In addition to exposures of organisms to ionizing radiation from a natural background, which is an unavoidable feature of life on Earth (7), low-dose/dose rate radiation exposure of the human population occurs during deep space flights (where astronauts are exposed to low- and high-LET radiation, mainly protons and high energy ions), clinical diagnostics, such as X-ray radiography and computed tomography, continuous nuclear work, or after nuclear accidents (8–10).

It is known that at the same dose, the cellular damage induced by high-LET radiation, for example, alpha particles, is different from that induced by low-LET radiation, for example, gamma rays. The latter mostly produce simple DSBs, which are repaired more effectively and faster than more complex, clustered DNA damage produced by alpha particles (6, 11, 12). Moreover, the quantitative outcome of cellular or chromosomal damage per unit dose at acute exposure differs from that of protracted irradiation at a low dose rate. At the same dose, acute gamma irradiation induces damage with a higher frequency compared with that induced by protracted gamma irradiation at a low dose rate, suggesting that low-dose rate radiation-induced damage is repaired efficiently and correctly with a system that was relatively error-free compared to that repairing damage caused by high dose rate irradiation (13–15). However, some studies have reported an inverse dose-rate effect for cataracts associated with low-LET radiation occupational exposures and in the epithelial cells of the mouse eye lens after γ-radiation exposure (16–18).

Moreover, in the range of low dose/dose rate exposures, several bodies of experimental evidence have shown the presence of non-targeted effects, i.e., effects arising in cells that are not directly irradiated, like bystander effect and genomic instability (19–22). The molecular mechanisms activated in response to chronic irradiation could differ from those activated in acute irradiation. The latter are fairly well elucidated, while little is known about the mechanisms activated in response to chronic exposure (23). The effects of low dose and low dose rates are certainly the result of complex network responses (that include genetic, epigenetic, metabolic, and immunological regulation) that have yet to be studied. *In vitro* radiobiology studies can provide a mechanistic understanding of the effects of low-dose radiation on cells, tissues, organs, and organisms and can complement epidemiological studies (24, 25).

To account for the effects of dose and dose rate of low-LET radiation, a single dose and dose rate effectiveness factor (DDREF) is currently applied for radiological protection to reduce cancer risks (26). However, the evidence base for this judgment continues to be debated (27). The greatest uncertainty arises from the relative lack of epidemiology data for low-dose/dose rate exposures. The gaps in knowledge regarding the biological effects of chronic irradiations should be filled, as humans and organisms are exposed to a low dose/dose rate of ionizing radiation every day (28). Cell and molecular data can potentially help to reduce this uncertainty. Several studies deal with the effects induced by acute irradiation of high and low-LET radiations on *in vitro* biological systems (29). Fewer studies are available on the effects induced by chronic low- or high-LET radiation exposure on these systems (the majority of them have mainly been performed using a dose rate >100 mGy/h) (27).

To the best of our knowledge, no studies have been performed comparing the effects induced on the same biological system in parallel by low- and high-LET radiations, imparted chronically and acutely (with dose rates as low as ~18 and 5 mGy/h). Thus, we decided to perform a radiobiological study with gamma rays (low LET) and alpha particles (high LET) in either chronic or acute conditions. Normal human fibroblasts were considered in our study as a valid model system of normal cell radiosensitivity to analyze the early and long-term effects of ionizing radiation (25). AG1522 normal human skin fibroblasts in the confluent state were irradiated using gamma rays from ^137^Cs or alpha particles with an average LET value of ~121 keV/μm for doses between 0.006 and 2.3 Gy. For chronic exposures, the same dose rate values of 18 and 5 mGy/h were considered for both low- and high-LET radiations, while for acute exposures, dose rate values of ~0.65 and 0.08 Gy/min were used for gamma rays and alpha particles, respectively. We tested the efficacy of such radiations to induce cytogenetic damage in terms of micronucleus formation (which is a well-known and widely used quantitative, dose-dependent biomarker of radiation impact at a cellular level) and overall post-radiation cell survival.

## 2 Materials and methods

### 2.1 Cell culture

AG1522 normal human foreskin fibroblasts were obtained from the Genetic Cell Repository at the Coriell Institute for Medical Research (Camden, NJ). The cells were cultured as a monolayer, with a doubling time of 24 h at 37°C in humidified air containing 5% CO_2_. They were grown in Alpha Minimum Essential Medium supplemented with 20% fetal bovine serum (FBS), 2% l-glutamine, 1.4% HEPES buffer solution (1 M), and 1% penicillin and streptomycin (all reagents from Euroclone S.p.A.). All experiments were conducted using cells from passages 9–16.

For gamma and alpha-particle irradiation experiments, cells were seeded in 12.5 cm^2^ polystyrene flasks or 9.6 cm^2^ stainless steel dishes with 2.5-μm-thick Mylar foil bottoms, respectively, at a seeding density of ~3.5 × 10^5^ cells per dish. Experiments began 96 h after the last feeding in confluent cultures. The cell sheet was examined microscopically for confluency using a Leica DM IL microscope with 10×, 20×, and 40× objectives. During irradiation (which lasted up to 12 days), the cells were maintained without changing the medium.

### 2.2 Irradiations

Irradiations with both gamma rays and alpha particles were performed at the Istituto Superiore di Sanità (ISS), Rome, Italy. Irradiations with alpha particles were performed using an irradiator previously described (30–32). Briefly, the irradiator is made of a stainless-steel cylinder inserted into a standard cell culture incubator. The top flange has a hole where a specially designed Petri dish can be inserted. This latter is made of stainless steel with a 2.5-μm-thick Mylar bottom of 56 mm inner diameter where cells grow as a monolayer. The chamber, equipped with ^241^Am or ^244^Cm sources, was filled with helium gas to reduce the energy loss of alpha particles emitted by the source. Cells were exposed to alpha particles from a ^241^Am source (≈ 3.6 MBq) for the acute irradiations and a ^244^Cm source (≈ 0.07 MBq) for the chronic irradiations. The dose rate was ~4,980 mGy/h for the acute irradiations and ~18 mGy/h and 5 mGy/h for the chronic irradiations. A collimator placed on the ^244^Cm source was used to obtain the lowest dose rate. The distance between the ^241^Am and ^244^Cm sources and the cell sample was chosen to equal 6 and 14 cm, respectively, to have the same alpha particle average energy at the cell entrance of 3 MeV with FWHM (full width at half maximum) of 0.1 MeV both with and without the collimator. The corresponding incident average LET, calculated in MS20 tissue, was equal to 121 keV/μm for both the acute and the chronic irradiations. The dose was determined using the relationship:


(1) $$\begin{array}{l} D\left(Gy\right)=1.6\times 10^{-9}\times \rho^{-1}\times F\times L, \end{array}$$

where ρ is density (g^−1^ cm^−3^), *F* is fluence (cm^−2^), L is LET (kev μm^−1^), and 1.6 × 10^−9^ is the conversion factor (30, 31). In our study, the density was accepted to be ρ = 1 g^−1^ cm^−3^. The fluence *F* was measured using CR39 plastic track detectors, and the LET was determined by the measurement of energy at the position of the cell monolayer performed using an ion-implanted silicon charged-particle detector (ORTEC model BU-020-450-AS). In all cases, the dose variability of alpha particles on the sample was better than ±7%, and control cells (sham-irradiated) were considered in parallel with irradiated cells.

We calculated the average number of alpha tracks, *N*, traversing a mean nuclear area for a given dose, assuming a Poisson distribution of the number of tracks per cell nucleus according to the equation:


(2) $$\begin{array}{l} \;N=A\left({\mu m}^{2}\right)\times D\left(Gy\right)\times {\left[0.16\times L\left(keV\times {\mu m}^{-1}\right)\right]}^{-1}, \end{array}$$

where *A* is cell nuclear area (μm^2^), *D* is radiation dose (Gy), and *L* is LET (kev μm^−1^). The AG1522 cell nuclear area, *A*, was measured in confluent cultures. To this purpose, AG1522 cell nuclei were stained with DAPI (4′,6-Diamidino-2-Phenylindole, Dilactate) solution in Vectashield Mounting Medium (Vector Laboratories, Inc., Burlingame, CA) at room temperature for 5 min. Then, the images of cell nuclei were recorded by a digital camera, which was attached to a fluorescent microscope. After calibration, area A was measured using ImageJ software (https://imagej.net/ij/index.html) with a micrometer slide. According to the Poisson distribution, the probability *P* that *n* particles traverse a given nuclear area is given by:


(3) $$\begin{array}{l} P\left(n\right)\;=\;N^{n}\times e^{-\frac{N}{n}}!, \end{array}$$

where *N* is the average number of alpha tracks traversing a mean nuclear area. The fraction of non-hit cell nuclei is *e*^−*N*^, and the fraction of cell nuclei traversed by at least one particle is 1–e^−*N*^.

For irradiations with gamma rays, ^137^Cs sources were used [LET ~0.8 keV/μm (33)]. Precisely, cells were exposed for acute irradiations using a gamma irradiator (Gammacell 40, Nordion Inc., Ottawa, Canada) at a dose rate of 40,200 mGy/h. The source-to-cells distance was 40 cm, and the irradiated field size was 5 × 5 cm^2^. The dose distribution homogeneity across the irradiated area from the center to the field's periphery was better than 95%. For chronic irradiation, cells were irradiated inside a cell incubator using the LIBIS irradiation facility developed at the ISS (32). The flasks were placed at ~26 and 50 cm from the ^137^Cs source to obtain dose rates of ~18 and 5 mGy/h, respectively. The dose range was (0.006–0.9 Gy) for alpha particles and (0.4–2.3 Gy) for gamma rays. For chronic irradiation at 18 mGy/h, the total duration of exposures at the doses of 0.006, 0.1, 0.4, 0.9, 1.4, and 2.3 Gy was ~20 min, 6, 22, 50, 78, and 128 h, respectively. For chronic irradiation at 5 mGy/h, the total duration of exposures at the doses of 0.4, 0.9, and 1.4 Gy were ~80, 180, and 280 h, respectively. For acute irradiation, the total duration of exposure ranges from 1 min at the lowest doses to some minutes at the highest doses. Confluent cells were acutely irradiated in our irradiation scheme at the beginning of each chronic irradiation. In a preliminary experiment, cells were acutely irradiated with gamma rays at different confluence days to see if the time of the “confluence period” could affect the final results. For the various chronic irradiations, the “confluence period” time corresponded to the exposure time, and it was thus different for each dose rate. Controls were also considered at different confluence days to exclude that the length of the “confluence period” could impact the spontaneous MN frequency. Cells were maintained at 37°C during chronic irradiation in a humidified CO_2_ incubator.

### 2.3 Micronucleus assay

The presence of micronuclei (MN) was evaluated by cytokinesis-block micronucleus assay (34). Briefly, after irradiation, confluent AG1522 cells were dissociated by trypsinization, and ~1.7 × 10^5^ harvested cells were seeded in chamber flaskettes (SPL Life Sciences, Co. Ltd., Korea). Gentle pipetting was used to separate the cells and to distribute them evenly on the slides. After the chamber, flaskettes were incubated in a humidified incubator at 37°C with 5% CO_2_ for 24 h to allow the cells to attach to the bottom of the slides. Following the incubation, the cells were treated with cytochalasin-B (Sigma-Aldrich) at a final concentration of 1.5 μg/ml to block cytokinesis. Preliminary tests were previously carried out in our laboratory considering different treatment times with Cyt-B to maximize MN frequency; these tests obtained an optimal treatment time with cytochalasin-B of 72 h. Therefore, we incubated the cells for 72 h in the cell culture incubator to accumulate binucleated cells (BNC). After that, the cells were washed with PBS and fixed in ethanol/acetic acid 1:5 for 15 min; the cell nuclei were stained with DAPI solution in Vectashield Mounting Medium (Vector Laboratories, Inc., Burlingame, CA) for 15 min, and MN was scored in BNC and classified according to the standard criteria (35). At least 1,000 (BNC) for treatment were scored using the Metafer v 3.12.112 after modifying the classifier developed for selecting micronuclei in BNC to increase its sensitivity. This number of BNC has been usually stated as sufficient to reach a statistically significant difference between radiation-exposed samples and unirradiated control, considering the background yield of MN in human cells; this value was typically reported in the literature on the micronuclei tests, including the IAEA Manual on Cytogenetic Dosimetry (IAEA Report Number EPR-Biodosimetry-2011) and respective ISO (ISO 17099:2014), and many other publications presenting similar experimental data (15, 21, 23, 34). A semi-automated analysis mode has been implemented, i.e., after automatic scanning and machine detection of micronuclei, the image gallery of all BN cells was visually checked by an operator to correct any errors and eliminate false positives.

### 2.4 Evaluation of the nuclear division index

Mononucleated, binucleated, trinucleated, and tetranucleated cells were scored to calculate the nuclear division index (NDI) using the formula provided by Eastmond and Tucker (36):


(4) $$\begin{array}{l} NDI=\left\{\left[M1+\left(2\times M2\right)+\left(3\times M3\right)+\left(4\times M4\right)\right]\times N^{-1}\right\}, \end{array}$$

where M1, M2, M3, and M4 represent the number of cells with 1–4 nuclei, and *N* represents the total number of scored cells. For each experiment, at least 1,000 cells were scored.

### 2.5 Cell survival

For clonogenic cell survival experiments, after irradiation, confluent AG1522 cells were trypsinized, counted, diluted, and plated into four 10 cm dishes at the appropriate concentration (1,000–5,000 cells per dish for the different doses considered) to score a number of colonies ranging from 100–250 per dish. After ~12 days of growth in a humidified incubator at 37°C with 5% CO_2_, viable clones from three flasks were fixed, stained, and scored for early survival evaluation (colonies with more than 50 cells were considered survivors). For each experiment, a mean number of colonies from three or more Petri dishes was calculated for both unirradiated control and considered doses, and then the survival values were determined. Cell surviving fractions were evaluated as the mean from at least three independent experiments with its standard error at each experimental radiation dose point.

### 2.6 Statistical analysis

Data were presented using the mean ± standard error obtained from at least three independent experiments. Statistical differences between the results obtained from different treatments were analyzed using the student's *t*-test. Values of *p* < 0.05 were considered statistically significant.

The micronuclei distributions were analyzed to test for compliance with Poisson statistics using Papworth's *u*-test. The mean number of observed micronuclei per cell (*y*) was given for each dose (*D*) along with dispersion index (σ^2^/*y*) and *u*-test (*u*). The *u*-values were calculated using the relation:


(5) $$\begin{array}{l} u=\left(\frac{\sigma^{2}}{y}-1\right)\sqrt{\frac{N-1}{2\left(1-\frac{1}{X}\right)}}, \end{array}$$

where *N* indicates the number of cells analyzed, and *X* is the number of micronuclei detected. The variance ^2^ was calculated by the relation:


(6) $$\sigma^{2}=\frac{{\left(0-y\right)}^{2}N_{0}+{\left(1-y\right)}^{2}N_{1}+{\left(2-y\right)}^{2}N_{2}+\ldots +{\left(i-y\right)}^{2}N_{i}}{(N_{0}+N_{1}+N_{2}+\ldots +N_{i})-1}$$

where *N*_0_, *N*_1_, *N*_2_…*N*_i_ refers to the number of cells carrying 0, 1, 2,…*i* micronuclei, respectively. Positive u values above 1.96 indicate overdispersion (at 95% confidence level) compared to perfect Poisson distribution (*u* = 0).

For the dose-response curves of micronuclei yield, data were fitted with a linear relationship:


(7) $$\begin{array}{l} y=C+\alpha \times D, \end{array}$$

where *D* is the absorbed dose, *C* is the background frequency, and α is the linear coefficient. For the dose-response curves of cell survival, data were fitted by a straight line on a semi-logarithmic plot:


(8) $$\begin{array}{l} S\;\left(D\right)=exp\left(-\alpha \times D\right), \end{array}$$

where *D* is the absorbed dose, and α is the linear coefficient. The data were fitted using the weighted Least Squares method. The goodness of fits was estimated using the (χ^2^) test and the *F*-test, and the errors represent the standard error values of the coefficients. A Pearson correlation analysis was performed to investigate the correlation between clonogenic cell survival and cytogenetic damage. We used a *t*-test to evaluate *p*-values for correlation significance with *t* = r^*^df^1/2^/(1 – *r*^2^)^1/2^, where df is the number of degrees of freedom and *r* is the Pearson correlation coefficient.

## 3 Results

### 3.1 Traversals of alpha particles through the mean nuclear area

**Figure 1:**
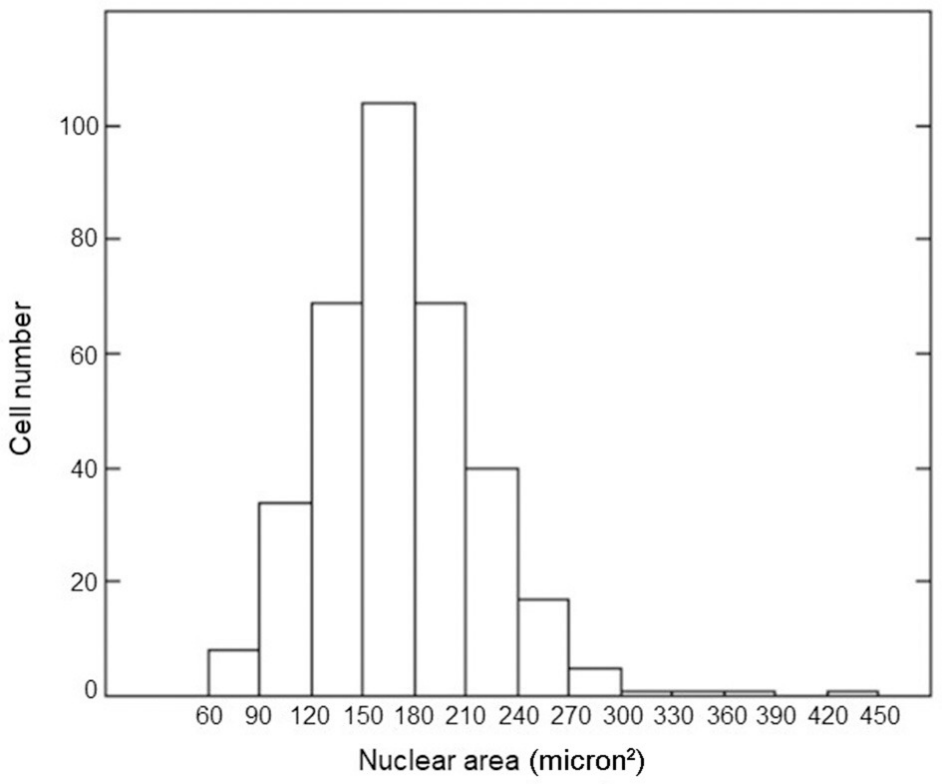
Histogram of the nuclear areas of confluent AG1522 cells.

We measured the nuclear area for 350 cells in confluent cultures through appropriate calibration using the ImageJ program. The distribution of nuclear areas is shown in Figure 1. The average value of the nuclear area was equal to 158 μm^2^ with a standard deviation of 47 μm^2^. This value agreed with the literature data (37–39). The average number of alpha tracks traversing this mean nuclear area and the fraction of cellular nuclei receiving 0, 1, more than one, and at least one alpha traversal as a function of considered doses were calculated by Equations 2, 3; the results are shown in Table 1. For the lower dose considered in our experiment, most of the cellular nuclei (~95%) were not traversed by alpha tracks; for the higher dose, the vast majority (more than 99%) of the cellular nuclei were traversed by an average of one or more alpha tracks.

**Table 1:** Estimates of alpha particle traversals per nucleus when confluent AG1522 cells are exposed to different mean absorbed doses.

**Dose (Gy)**	**Average number of traversals per nucleus**	**Fraction of cell nuclei traversed by 0, 1, more than 1, and at least one alpha particle**			
		***P*** **(0)**	***P*** **(1)**	***P*****(** > **1)**	***P*****(** > **0)** = **1 –** ***P*****(0)**
0.006	0.05	0.952	0.047	0.001	0.048
0.1	0.82	0.443	0.361	0.196	0.557
0.4	3.26	0.038	0.125	0.837	0.962
0.9	7.34	0.001	0.005	0.994	0.999

The calculations were based on the measured average nuclear area of 158 μm^2^.

### 3.2 Micronuclei

Cytogenetic damage was studied by analyzing the induction of MN in AG1522 cells chronically and acutely exposed to gamma rays and alpha particles, where a dose rate of 18 mGy/h was chosen for chronic irradiation. For chronic irradiation, the exposure time ranges from a few hours to several days, depending on the dose value. In a preliminary experiment, cells were acutely irradiated with gamma rays on the first confluence day at doses of 0.9 Gy and 1.4 Gy, on the seventh confluence day at a dose of 0.9 Gy, and on the 12th confluence day (time of the longest “confluence period” in our experiments) at a dose of 1.4 Gy. In contrast, control (non-irradiated) cells were considered on the 1st and 12th days of confluence. The micronucleus frequency values obtained after these irradiations were very close (at the same dose); the micronucleus frequency values of the controls on day 1 and day 12 of confluence were also very close (see Supplementary Table 1). This suggests that the time of the ‘confluence period' does not affect the final results. Therefore, we decided to perform the acute exposures at the beginning of the ‘confluence period' in all experiments. The controls were always considered at the beginning of the “confluence period.”

**Figure 2:**
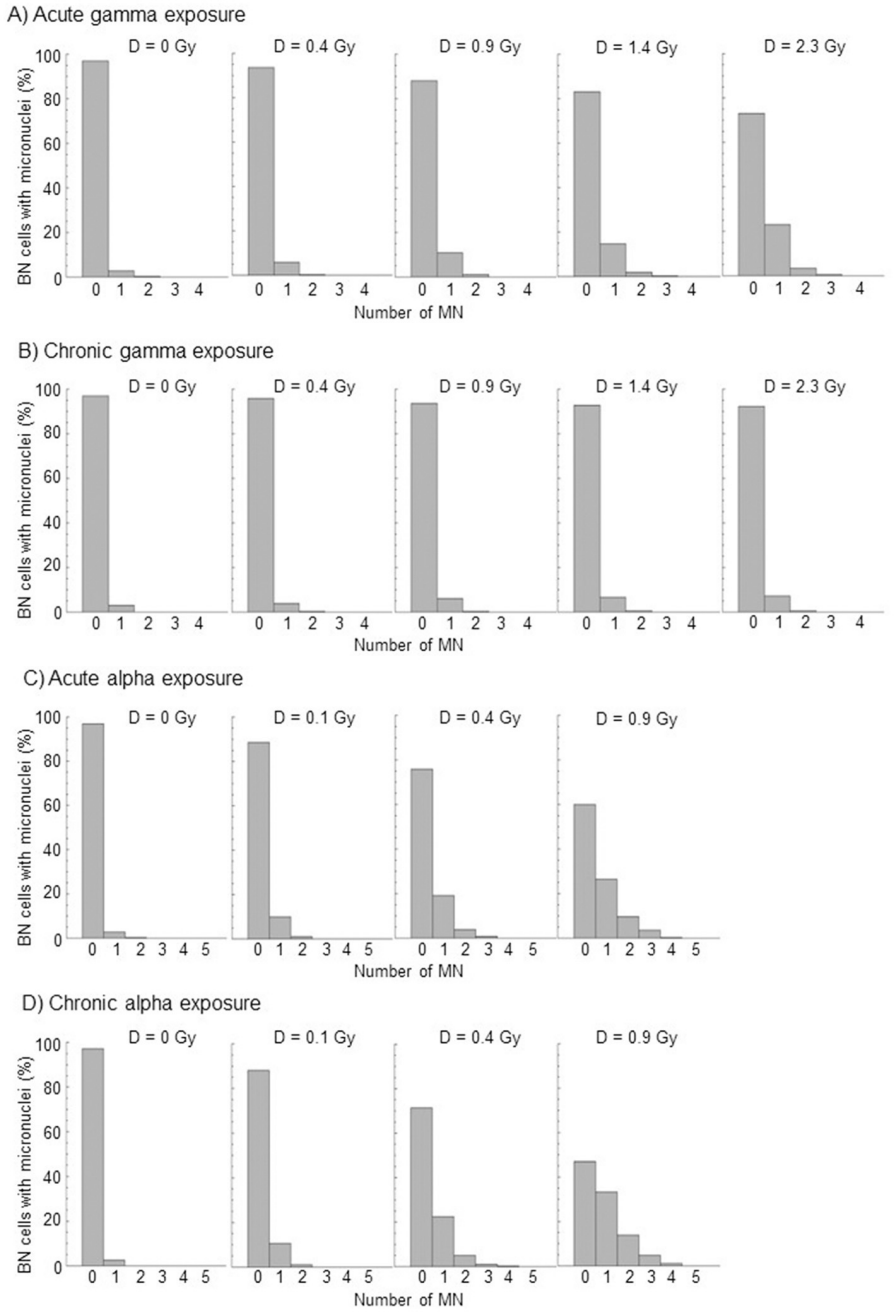
Distributions of micronuclei, relative to the total numbers of BN cells scored, by acute and chronic gamma and alpha irradiation from at least three independent experiments. **(A)** Acute gamma exposure, **(B)** Chronic gamma exposure, **(C)** Acute alpha exposure, and **(D)** Chronic alpha exposure.

The cell distribution of MN at each dose of acute and chronic gamma and alpha irradiation from at least three independent experiments is shown in Figure 2. Table 2 summarizes the total number of BN cells scored, number of MN observed, dispersion index (σ^2^/*y*), and *u*-test (*u*) for compliance with Poisson statistics. As expected for micronuclei, the data tend to overdispersion with respect to the Poisson distribution at all doses, even with photon irradiation. This overdispersion was greater for alpha particles than for gamma rays.

**Table 2:** Dispersion index (σ^2^/y) and u-test (u) for compliance with Poisson statistics evaluated by the distributions of micronuclei by acute and chronic gamma and alpha irradiation.

**Dose (Gy)**	***N***	***X***	**σ^2^/*y***	***u***
**(A) Acute gamma**				
0	4,311	139	1.10	4.54
0.4	5,269	369	1.14	7.28
0.9	5,559	742	1.09	4.63
1.4	5,092	998	1.11	5.59
2.3	5,012	1,589	1.03	1.53
**(B) Chronic gamma**				
0	4,866	158	1.04	2.16
0.4	4,120	183	1.10	4.46
0.9	3,730	260	1.08	3.32
1.4	4,317	342	1.08	3.94
2.3	5,793	487	1.05	2.56
**(C) Acute alpha**				
0	4,313	135	1.10	4.77
0.1	5,364	685	1.14	7.46
0.4	5,375	1,618	1.19	9.72
0.9	2,255	1,309	1.21	6.90
**(D) Chronic alpha**				
0	3,887	111	1.08	3.53
0.1	6,999	948	1.11	6.60
0.4	4,768	1,740	1.16	7.67
0.9	3,393	2,691	1.07	2.86

N, total numbers of BN cells scored; X, numbers of MN observed.

**Figure 3:**
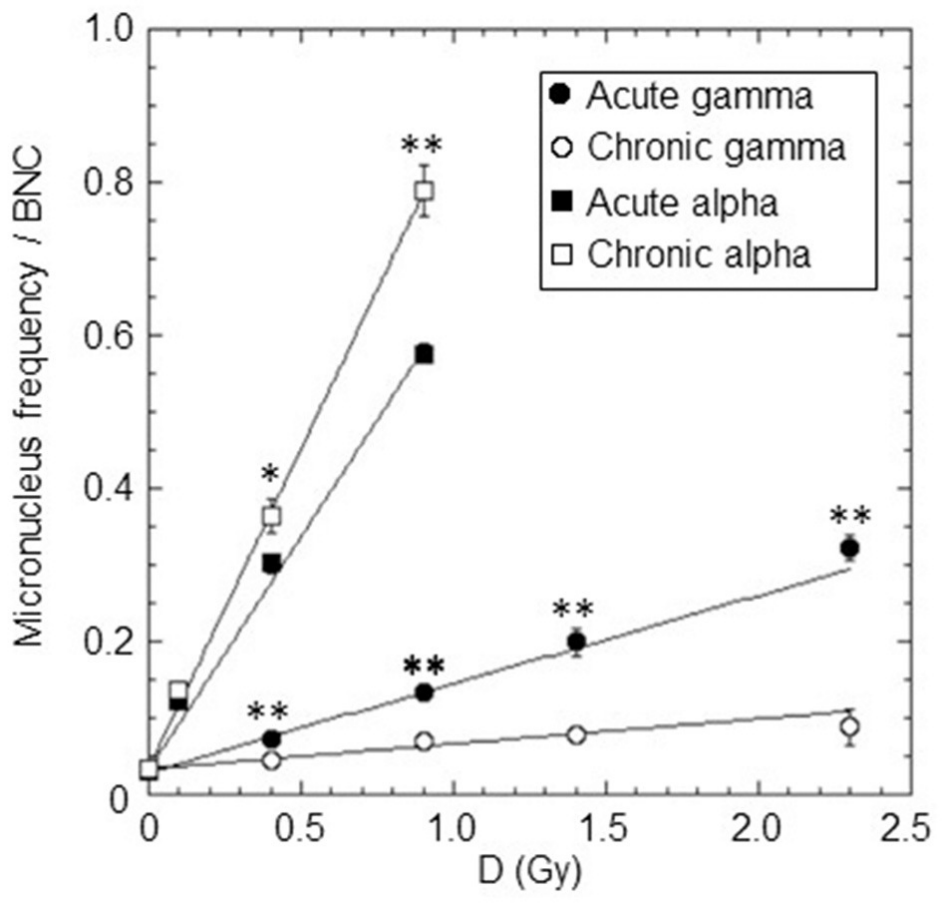
Dose-response curves for the micronuclei yield (micronucleus frequency/BNC vs. dose) in confluent AG1522 cells irradiated with different doses of gamma rays (0.4, 0.9, 1.4, and 2.3 Gy) and alpha particles (0.1, 0.4, and 0.9 Gy), at a high dose rate (of ~40,200 and 4,980 mGy/h for gamma rays and alpha particles, respectively) and at a low dose rate (~18 mGy/h for both gamma rays and alpha particles). Data points represent the means of at least three independent experiments with ~1,000 BNC scored per experiment; the error bars represent the standard error of the mean. The statistical difference between the results obtained at the same dose for chronic and acute gamma exposures was analyzed using the student's *t*-test. The statistical difference between the results obtained at the same dose for chronic and acute alpha exposures was analyzed using the student's *t*-test. Values of *p* < 0.05 (*) and *p* < 0.01 (**) were considered as statistically significant.

The micronuclei yield, *y*, was evaluated by averaging micronucleus frequency per BNC from at least three independent experiments for each dose and type of radiation. The dose-response curves for cytogenetic damage, expressed as micronucleus frequency/BNC vs. dose, in confluent AG1522 cells, acutely and chronically irradiated with different doses of gamma rays (in the range 0.4–2.3 Gy) and alpha particles (in the range 0.1–0.9 Gy), were shown in Figure 3. For both gamma rays and alpha particles, the data obtained were fitted with a linear relationship (see Equation 7). The values of the coefficients C and α obtained from the fit and the number of degrees of freedom df are shown in Table 3. The *p*-values of the χ^2^ test shown in Table 3 confirm a good fit. Moreover, the significance of the linear coefficient α was also confirmed by the *F*-test (the ratio between the coefficient and its SE); for gamma-ray irradiations, the *F*-value was higher than 9.28 [the cutoff value for *F*_0.05_ (3, 3)], and for alpha particle irradiations, the *F*-value was higher than 19.00 [the cutoff value for *F*_0.05_ (2, 2)]. The relative biological effectiveness (RBE) for micronucleus induction by alpha particles compared to acute and chronic gamma radiation was calculated from the ratio of the α-coefficients and was also reported in Table 3. These data showed that the gamma-ray exposure of AG1522 cells at high dose rates was more effective in leading to the induction of MN than the same exposure delivered at a low dose rate. Comparisons were made at the same dose between chronic and acute gamma exposures, and significant differences were obtained (*p* < 0.01, Student's *t*-test).

**Table 3:** *C*, α, and RBE values for micronuclei induction by acute and chronic gamma and alpha irradiation.

**Radiation type**	***C* ±SE**	**α (Gy^−1^) ±SE**	**λ^2^**	**df**	**RBE_γ*Acute*_**	**RBE_γ*Chronic*_**
Acute gamma	0.029 ± 0.004	0.115 ± 0.008	2.24	3	–	–
		*F* = 12.78	*p* = 0.52			
Chronic gamma	0.034 ± 0.001	0.033 ± 0.003	2.68	3	–	–
		*F* = 11.00	*p* = 0.44			
Acute alpha	0.032 ± 0.003	0.611 ± 0.019	1.16	2	5.3 ± 0.4	18.5 ± 1.8
		*F* = 27.77	*p* = 0.56			
Chronic alpha	0.034 ± 0.003	0.835 ± 0.014	0.23	2	7.3 ± 0.5	25.3 ± 2.3
		*F* = 55.67	*p* = 0.89			

Values of degrees of freedom df and λ^2^ were reported together with p and F values.

Conversely, for alpha particles, low and high-dose-rate exposures were equally effective in inducing cytogenetic damage at the 0.1 Gy dose, while, surprisingly, for higher doses, chronic exposure was more effective than acute. Comparisons were made at the same dose between chronic and acute alpha exposures, and significant differences were obtained for 0.4 Gy (*p* < 0.05, Student's *t*-test) and 0.9 Gy (*p* < 0.01, Student's *t*-test). As expected, the exposure to alpha particles at both high and low dose rates was much more effective than the gamma-ray exposure for this endpoint.

The percentage of BNC with micronuclei, BNMN, in confluent AG1522 cells irradiated with a very low dose of alpha particles equal to 0.006 Gy, delivered only at a low dose rate of 18 mGy/h, was also measured. We could not accurately deliver this dose at a high dose rate (4,980 mGy/h) since it corresponds to an irradiation time too short (~4 s). Three independent experiments were performed at 0.006 Gy (each with the correspondent control cells), and the mean percentage of BNMM obtained was slightly higher than that of the control cells (4.40 ± 0.10 vs. 3.30 ± 0.14). However, statistical analysis revealed that the difference between these two values was significant (*p* < 0.05).

**Figure 4:**
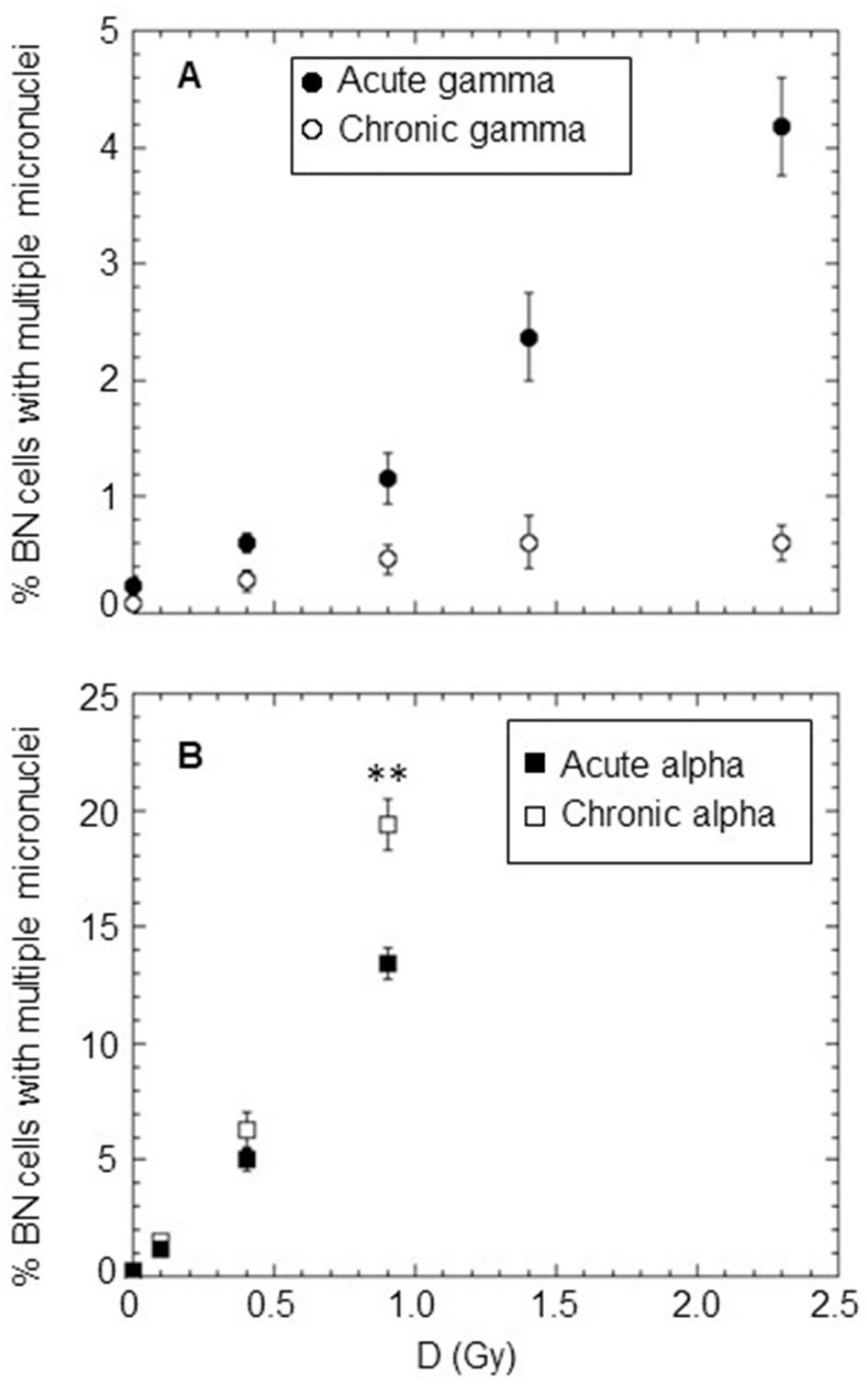
Percentage of BN cells carrying ≥2MN after acute and chronic treatments with different types of radiation: **(A)** gamma rays at a high dose rate of ~40,200 mGy/h (solid circle) and at a low dose rate of ~18 mGy/h (open circle); **(B)** alpha particles at a high dose rate of ~4,980 mGy/h (solid square) and at a low dose rate of ~18 mGy/h (open square). Each datum point represents the mean of at least three independent experiments with ~1,000 BNC scored per experiment; the error bars represent the standard error of the mean. The statistical difference between the results obtained at the dose of 0.9 Gy for chronic and acute alpha particle exposures was analyzed using the Student's *t*-test. A value of *p* < 0.01 (**) was considered statistically significant (Student's *t*-test).

The percentage of BNC carrying multiple MN (≥2 MN) was also determined for doses in the range of 0.1–2.3 Gy to obtain information about the severity of the induced cytogenetic damage by the different types of radiations (see Figure 4). The percentage of BN cells with more than two MN was very close to zero for the investigated doses of chronically delivered gamma rays. For acute gamma exposure, this percentage was higher than that of chronic gamma irradiation at the same dose, reaching a value of ~4% at the highest dose of 2.3 Gy (Figure 4A). The situation was completely different for alpha particles, where the percentage of BN cells with more than two MN was different from zero for both chronic and acute irradiation, increasing with dose (up to a value >10% at 0.9 Gy) Figure 4B. Very close values were obtained for chronic and acute treatments at the doses of 0.1 and 0.4 Gy (open and solid squares are superimposed for 0.1 Gy). However, surprisingly, at the dose of 0.9 Gy, the percentage of BN cells carrying multiple MN was significantly higher for chronic irradiation than acute irradiation. As expected, at the same dose, we found that alpha particles caused a much higher number of multiple micronuclei than gamma rays regardless of the considered dose rate, indicating a much more severe cytogenetic burden with multiple aberrations occurring in particular cells in the case of alpha particle exposure.

**Figure 5:**
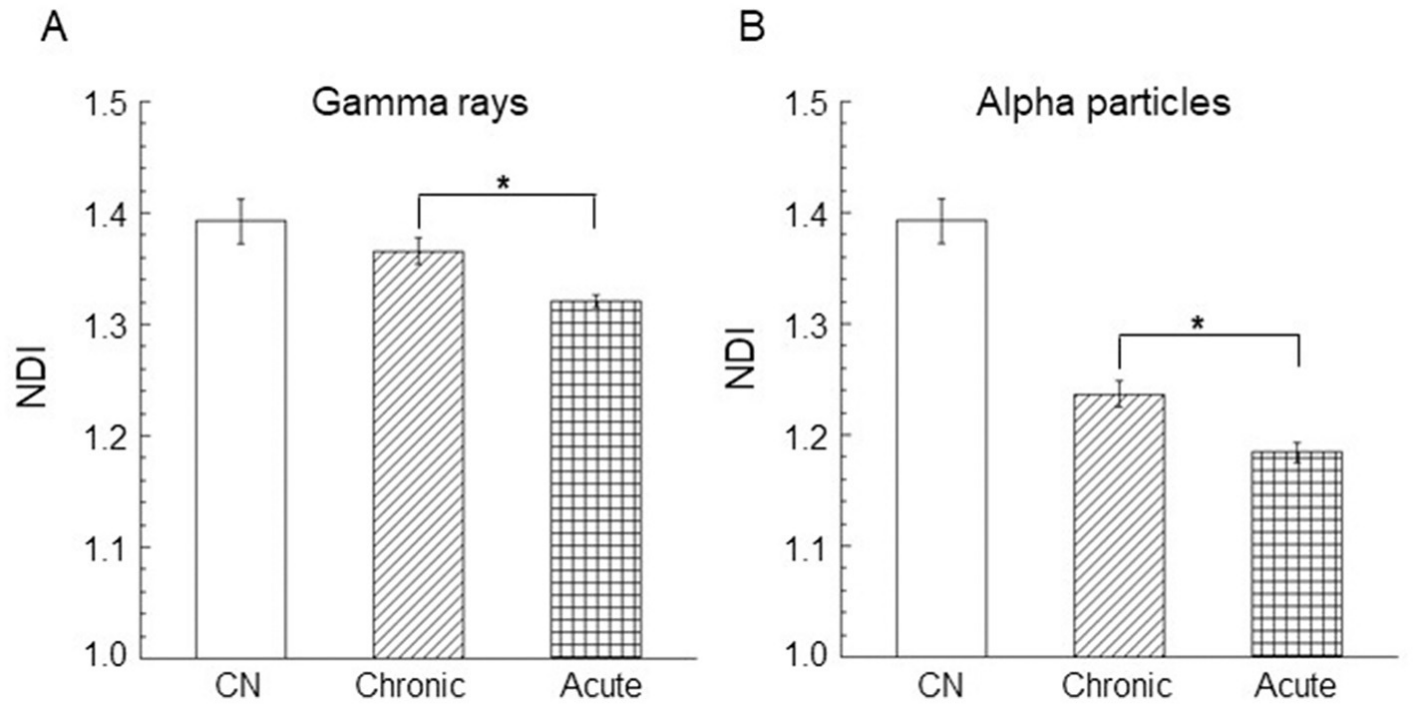
Nuclear division index (NDI) in AG1522 cells irradiated in the confluent state with a dose of 0.9 Gy of **(A)** gamma rays and **(B)** alpha particles at both low and high dose rates. Data represent the means of at least three independent experiments with at least 1,000 BNC scored per experiment; the error bars represent the standard error of the mean. The statistical differences between the results obtained for acute and chronic gamma exposures **(A)** and for chronic and acute alpha particle exposures **(B)** were analyzed using the student's *t*-test. A value of *p* < 0.05 (*) was considered statistically significant (student's *t*-test).

**Figure 6:**
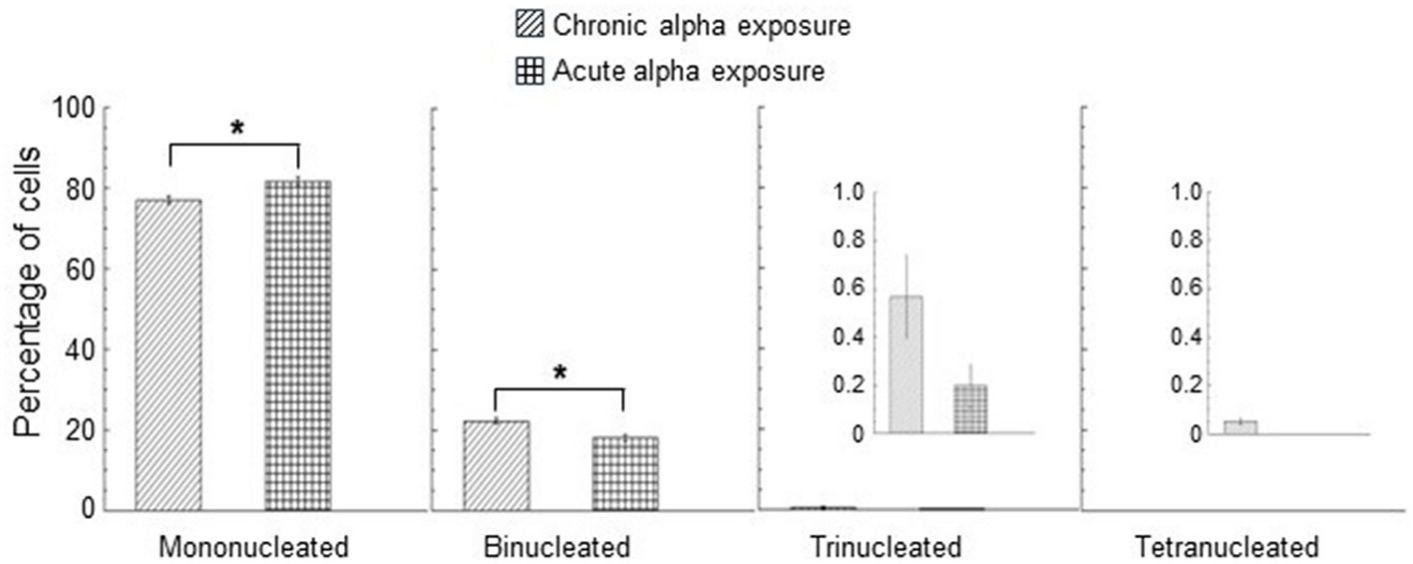
Percentage of mono/bi/tri/tetra-nucleated cells for chronic and acute alpha exposure at 0.9 Gy. A smaller y-scale insert was added to the percentage of trinucleated and tetranucleated cells. No tetranucleated cells were observed for acute alpha exposure. Data represent the means of at least three independent experiments with at least 1,000 BNC scored per experiment; the error bars represent the standard error of the mean. The statistical differences between the results obtained for mononucleated cells and for binonucleated cells were analyzed using the student's *t*-test. A value of *p* < 0.05 (*) was considered statistically significant (student's *t-*test).

The nuclear division index (NDI) was calculated in control cells (unirradiated cells) and only at the dose of 0.9 Gy for acute and chronic gamma ray and alpha particle irradiations. Three independent experiments were considered, and at least 1,000 cells were scored for each experiment. Mean NDI values, with standard errors, were shown for gamma rays, Figure 5A, and for alpha particles, Figure 5B. The percentage of mono/bi/tri/tetra-nucleated cells for chronic and acute alpha exposure is shown in Figure 6. An NDI value that was very close to that of control cells was obtained for chronically irradiated cells with gamma rays.

Moreover, a significantly lower NDI value was found in cells exposed to high dose rates with respect to the cells irradiated to low dose rates of gamma rays (*p* < 0.05). These results indicated that gamma acute exposure caused a small but significant reduction in cell proliferation activity compared to chronic exposure at a dose of 0.9 Gy. A more marked reduction in the cell proliferation activity was found for alpha particles than that induced by gamma rays. A significant difference was found between the values of the percentage of mononucleated cells for acute and chronic alpha exposures. The values of the percentage of binucleated cells were significantly higher for chronic alpha irradiation than for acute alpha irradiation (Figure 6). Moreover, the difference between the NDI values for acute and chronic alpha particle exposure, albeit small, was significant (*p* < 0.05), indicating a higher effect of the cell proliferation activity for acute vs. chronic irradiation (Figure 5B).

**Figure 7:**
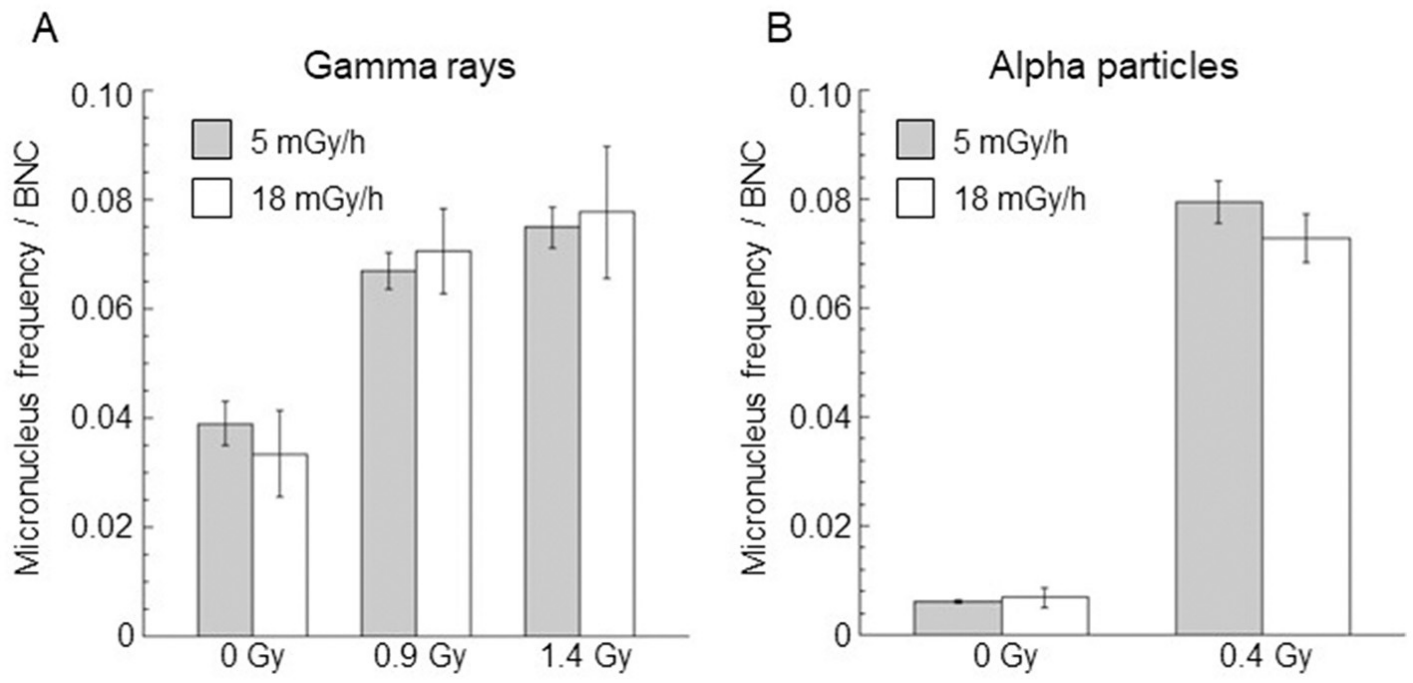
Comparison of micronucleus frequency per BNC induced by gamma rays **(A)** and alpha particles **(B)** at the same dose and for dose rates of 5 mGy/h (gray bar) and 18 mGy/h (white bar). Control values (not irradiated samples) were also reported. Data points represent the means of two independent experiments with ~1,000 BNC scored per experiment; the error bars represent the standard error of the mean.

A dose rate of < 18 mGy/h was also considered to verify possible cytogenetic damage changes as the chronic irradiation dose rate changed. In particular, micronucleus frequency per BNC in confluent AG1522 cells irradiated at a dose rate of 5 mGy/h, with doses of 0.9 Gy and 1.4 Gy for gamma rays and 0.4 Gy for alpha particles, was evaluated. The results are shown in Figure 7, where the data obtained at 18 mGy/h were also reported for comparison. Micronucleus frequency per BNC obtained at 5 mGy/h was not significantly different from those observed at 18 mGy/h for both gamma and alpha particles.

### 3.3 Cell survival

**Figure 8:**
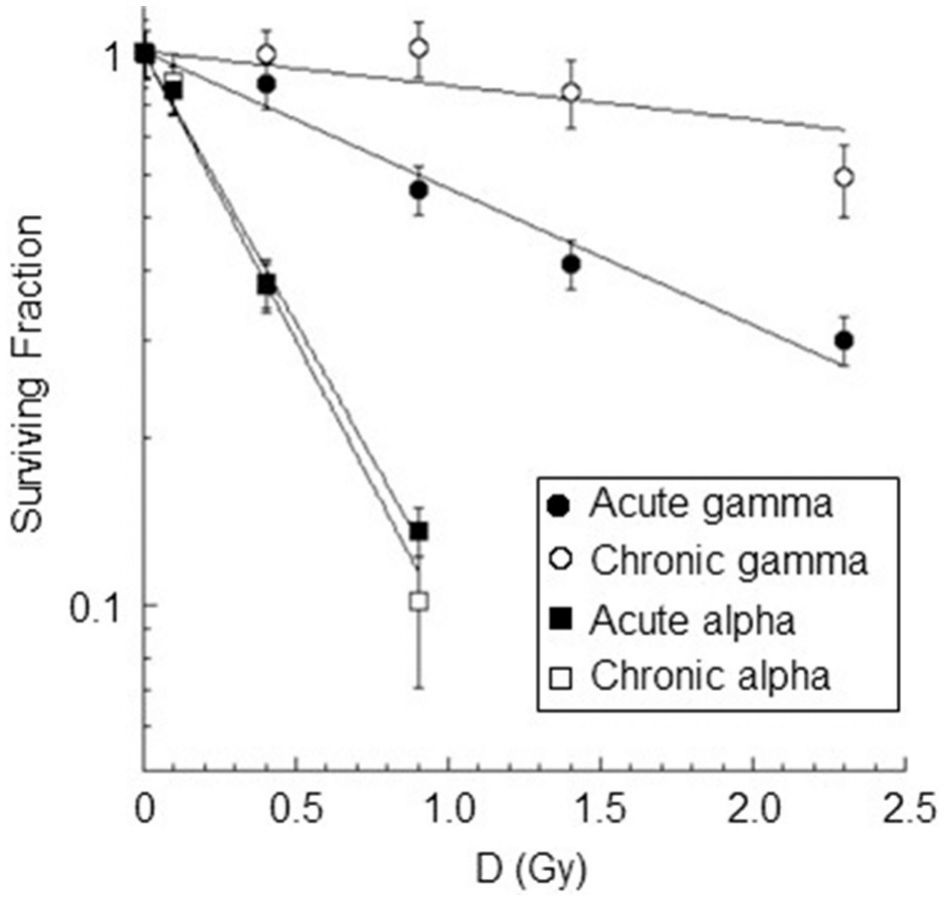
Dose-response curves for cell inactivation in confluent AG1522 cells after treatment with gamma rays (at doses of 0.4 Gy, 0.9 Gy, 1.4 Gy, 2.3 Gy) and alpha particles (at doses of 0.1 Gy, 0.4 Gy, 0.9 Gy) delivered chronically (at 18 mGy/h for both gamma rays and alpha particles) and acutely (at 40,200 mGy/h and 4,980 mGy/h for gamma rays and alpha particles respectively). Cells were trypsinized within 5–10 min after exposure. Each datum point represents the mean of at least three independent experiments, and the error bar denotes the standard error of the mean. The lines are the best fit for the data.

Survival curves for AG1522 confluent cells irradiated with different doses of gamma rays and alpha particles at low dose rates (18 mGy/h) and high dose rates (40,200 and 4,980 mGy/h for gamma rays and alpha particles, respectively) were shown in Figure 8. For chronic gamma exposure, a cell survival of ~100% was obtained up to a dose of ~0.9 Gy; for higher doses, a slight decrease in cell survival was observed up to a value equal to ~60% for a dose of 2.3 Gy. Table 4 shows the values of α, obtained from the best-fitting of the data reported in Figure 8. These data showed that the gamma-ray exposure of AG1522 cells at high dose rates was more effective in causing the reduction of the clonogenic cell survival than the same exposure delivered at a low dose rate. Instead, low and high-dose-rate exposures were equally effective for alpha particles in reducing the clonogenic cell survival to a dose of 0.9 Gy. Chronic exposure appeared to be more effective than acute exposure in inducing the reduction of the clonogenic cell survival for the dose of 0.9 Gy, but the difference in the measured values was not statistically significant. The exposure to alpha particles at both high and low dose rates was more effective than the gamma-ray exposure, which also caused the reduction of clonogenic cell survival. RBE values for both chronic and acute exposure were calculated as the ratio of α coefficients of the alpha particles and gamma rays. These values are shown in Table 4.

**Table 4:** Values of the linear coefficients α and RBE for cell inactivation by acute and chronic gamma and alpha irradiation.

**Radiation type**	**Irradiation**	**α (Gy^−1^) ±SE**	**RBE_γ*Acute*_**	**RBE_γ*Chronic*_**
Gamma rays	Acute	0.57 ± 0.06	–	–
	chronic	0.14 ± 0.02	–	–
Alpha particles	Acute	2.26 ± 0.10	4.0 ± 0.5	
	chronic	2.40 ± 0.11		17.1 ± 2.6

The RBE values were evaluated as the ratio of α coefficients of the alpha particles and gamma rays.

**Figure 9:**
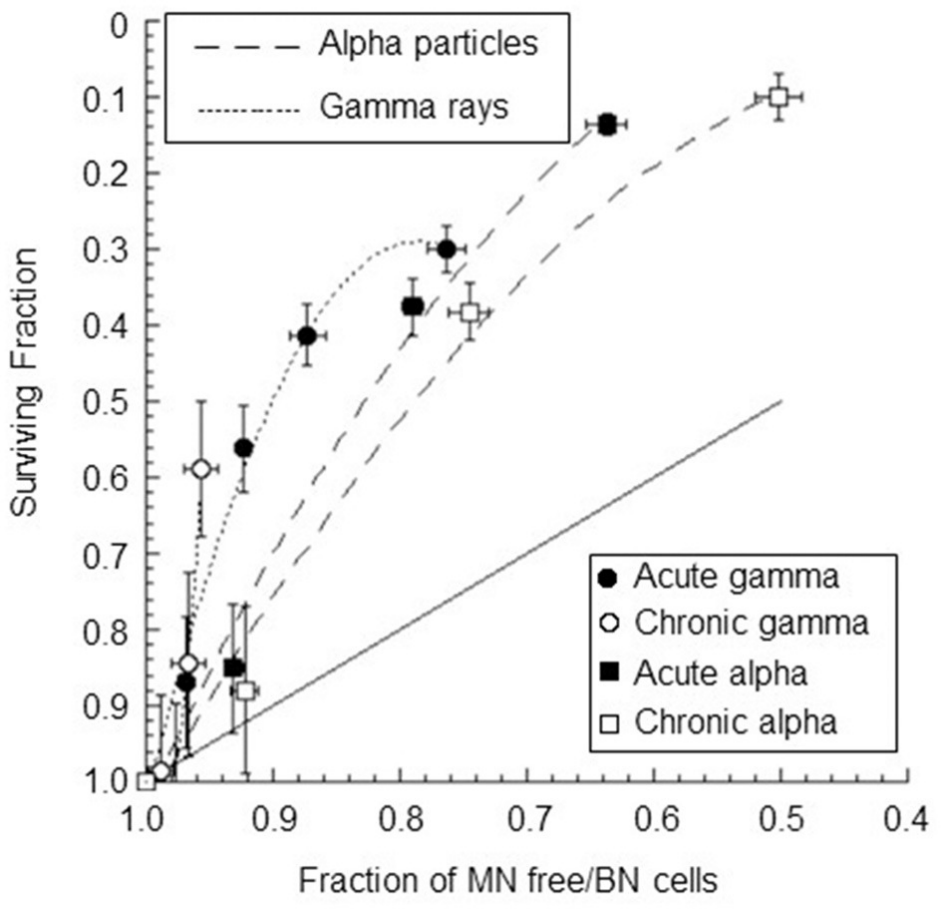
Surviving fraction plotted against the fraction of BNC without MN for confluent AG1522 cells after treatment with gamma rays and alpha particles delivered chronically (at 18 mGy/h for both gamma rays and alpha particles) and acutely (at 40,200 mGy/h and 4,980 mGy/h for gamma rays and alpha particles, respectively); the error bars represent the standard error of the mean. The dotted lines represent the polynomial curve fits of the data, and the solid line represents a one-to-one relationship.

The correlation between the surviving fraction and the fraction of cells without micronuclei was also studied (see Figure 9). A strong, significant positive correlation between the surviving fraction and the fraction of cells without micronuclei was observed for all types of radiation except for chronic gamma rays (*r* = 0.93±0.21 *p*-values of 0.02 for acute gamma rays, *r* = 0.83±0.32 *p*-values of 0.08 for chronic gamma rays, *r* = 0.99±0.11 *p*-values of 0.01 for acute alpha particles, and *r* = 0.98±0.14 *p*-values of 0.02 for chronic alpha particles). Notably, the fraction of cells without micronuclei was higher than the surviving fraction. For instance, at a 60% level of cell survival, the percentage of cells not expressing micronuclei was 96% for chronic gamma-ray exposure, 93% for acute gamma-ray exposure, 83% for chronic alpha particle exposure, and 87% for acute alpha particle exposure.

## 4 Discussion

The reason why biological effects induced by radiation at low dose rates have been investigated by fewer studies than those induced at high dose rates (37, 40–47) is mainly related to the difficulties inherent in carrying out robust experimentations with chronic exposures. Experiments with chronically delivered radiation beams are very laborious, and the results obtained may not be easy to understand. This is because the damage inflicted on cells after chronic exposure may be minimal compared to the damage received from endogenous sources. Therefore, it is advisable that the same operator can perform the experimental activities, minimizing differences in sample handling. The goal is to minimize operator-dependent non-biological variations; in fact, small variations in the measurement result could be introduced by a different sensitivity of the operators in handling the sample during the experimental protocol.

Furthermore, e.g., for the micronucleus test, although there are standardized scoring criteria, the results may be subject to small variations depending on the operators' subjective interpretation of the micronucleated cells. Moreover, the duration of the radiation exposure of the biological sample can be as long as several weeks. For such reasons, experimental design should be well conducted with appropriate statistical analysis and parallel controls always included (27).

In this article, we studied the biological effects induced by low and high dose rates in human fibroblasts, attempting to follow these guidelines. Normal human fibroblasts can provide important information in biodosimetry and radiotherapy regarding the late effects of irradiation (fibrosis, tissue necrosis) that often limit radiation dosage (48, 49). For simplicity, confluent cells were used. Confluent cells can be a representative model system of healthy cells in the body that are not proliferating. However, there are normal cell types in the body that are proliferating. For example, under physiological conditions, fibroblasts proliferate and thus enable cell turnover, or they intervene in an inflammatory process or regular wound healing. It would be interesting in the future to carry out studies on the biological effects induced by acute and prolonged exposure to ionizing radiation in proliferating cells. In this case, the variation in radiation sensitivity during the cell cycle will have to be considered.

### 4.1 Acute and chronic gamma-ray exposures

The MN yield observed in our study after acute gamma irradiation, of ~0.12 Gy^−1^, was in good agreement with those obtained in other *in vitro* studies for fibroblasts at similar radiation dose points. For instance, Słonina et al. found an MN yield of 0.10 Gy^−1^ considering MN/BNC values in human fibroblasts acutely irradiated with 200 kV X-ray (50). An MN yield of 0.14 Gy^−1^ was obtained by Litvinchuk et al., considering MN/BNC values of human fibroblasts acutely irradiated with gamma rays (51). The ratio of the α-coefficients for micronuclei induction by acute and chronic gamma irradiations was equal to ~3.5, reflecting the increased induction of micronuclei when radiation was administered acutely rather than chronically. Although these results refer to an *in vitro* system and are not directly transferable to humans, they would seem to suggest that a gamma dose exposure delivered at a low dose rate should result in a lower biological response (e.g., cancer induction) and, consequently, a reduced risk than the same dose delivered at a high dose rate. Our results are in agreement with the data mentioned in the article by Geard and Chen, who exposed AG1522 cells at dose rates of 600 and 1.3 Gy/min for chronic and acute irradiation (13) and with the data reported by Nakamura et al., where SuSa/T-n cells were exposed at dose rates of 18 and 2 Gy/min for the chronic and acute irradiation (15).

These results showed that temporally concentrated (high dose rate) or elongated (low dose rate) gamma-ray exposures might differently contribute to cytogenetic damage (caused by misrepair of DNA breaks or unrepaired DNA). Initial cytogenetic damage may be a consequence of the interaction of pairs of DSBs close to each other produced by different (independent) tracks (inter-track contribution) or along the same track (intra-track contribution) (52, 53). The track structure of photons mainly consists of secondary electrons, which are set in motion by primary photon interactions, and ionizations in the cell are mainly induced by these electrons (54, 55). In our experimental setup, cells grow and adhere to a polystyrene flask. Secondary electrons can be set in motion both inside the cell and in the thickness of the flask, and some of them can interact inside the nucleus, producing DNA damage. At the doses considered, hundreds or thousands of particles (secondary electrons) interact inside the nucleus, inducing DNA strand breaks (56–59). In the case of acute gamma-ray exposure (40,200 mGy/h), initial cytogenetic damage can be induced not only with an intra-track contribution but also with an inter-track contribution because the repair of DNA strand breaks in human fibroblasts occurs with half-life times of tens of minutes up to a 100 min (60–63). In fact, at the considered doses, the total exposure duration is equal to tens of seconds or, at most, a few minutes. The time interval between the interaction of one particle and the next is equal to a fraction of a second. Consequently, several hundred or thousands of particles interact inside the nucleus, and DNA strand breaks induced by different particles may interact before being repaired.

Meanwhile, for chronic gamma exposure (at 18 mGy/h), the inter-track contribution is small compared to the intra-track contribution. In fact, in this case, the hundreds or thousands of particles interacting inside the nucleus and inducing DNA strand breaks are temporally distant: at the doses considered, the total exposure duration is tens or hundreds of hours, and the time interval between the interaction of one particle and the next was in minutes. Consequently, the average number of particles interacting inside the nucleus, inducing DNA strand breaks, in a 100 min (time comparable with the repair half-time) is small (of the order of tens of particles). The probability that two breaks (produced by different particles) would be close enough to interact before being repaired is small. Moreover, simple damage induced by low-dose-rate radiation should be repaired efficiently and correctly with a relatively error-free system, as suggested by Nakamura et al. (15). This leads to a reduction of the probability of misrepair, reducing the micronucleated cells (64). To understand the weight of the inter-track contribution for chronic gamma exposure, we further reduced the gamma dose rate to the value of 5 mGy/h (with a consequent increase in the mean time interval between the arrival of one interacting particle and the next by a factor of ~4), obtaining that the cytogenetic damage does not vary significantly from that obtained at 18 mGy/h at the same dose. This result seems to indicate that both exposures at 18 mGy/h and 5 mGy/h occur with a time course of induction of DNA strand breaks such that all induced reparable lesions are repaired before interacting with each other, giving rise to irreparable lesions that can be subsequently fixed and give rise to micronuclei (i.e., inter-track contribution is absent). The only irreparable lesions are those induced by single tracks (intra-track contribution), which are the same for the same dose at 18 mGy/h and 5 mGy/h. Therefore, below 18 mGy/h, the cytogenetic damage in confluent AG1522 cells depends only on the total dose, not the dose rate.

For chronic gamma exposure, the fraction of dead cells was very close to zero up to the dose of 0.9 Gy, although a fraction of ~2% micronucleated cells was observed at such a dose. This is consistent with the fact that not all micronucleated cells go against death by apoptosis or senescence, but some of them might survive and produce daughter cells with and without micronuclei, leading to genomic instability (65, 66). The indication of very slight cell damage induced by chronic gamma irradiation at 0.9 Gy is confirmed by the observation that no cell cycle delay was produced for this dose. Cell survival data also showed that acute gamma-ray exposure was more effective in cell killing than the same exposure delivered chronically. To quantify this higher effectiveness at low doses, we considered the ratio of α coefficients of the chronic and acute gamma exposures (see Table 4). The value obtained for this ratio was 4.1, indicating that at low doses, gamma acute exposure was approximately four times more effective in cell killing than gamma chronic exposure. Moreover, from the measured survival values, we also calculated the ratio:


$$\begin{array}{l} \frac{dead\;cells\;fraction\;per\;unit\;dose\left(2.3\;Gy,\;0.67\;Gy/min\;\right)}{dead\;cells\;fraction\;per\;unit\;dose\left(0.4\;Gy,\;18\;mGy/h\right)} \end{array}$$

i.e., the ratio between the fractions of dead cells evaluated at the highest dose and rate values and at the lowest values considered, obtaining a value of 8.8. This ratio takes into account the lower effectiveness of low-dose and low-dose-rate exposures. This difference may be due to sublethal damage (SLD) repair during chronic gamma-ray exposure. SLD is damage that can be repaired in a matter of hours when no additional sublethal damage is added, which could lead to lethal damage (67). SLD repair (SLDR) can occur in the time interval between doses in a split-dose experiment or during exposure in a low-dose-rate experiment, and it is considered to represent the repair of DSB. SDLR during the course of protracted irradiation is a very important factor governing the dose-rate effect. In this case, the number of sites of SLD present at any one time will be reduced, decreasing the probability of interaction and, therefore, the biological effect for a given dose. Liu et al. (68) have shown that Ku-dependent classical non-homologous end-joining is the main pathway for SLDR in mammalian cells (including human cells).

The survival fraction and the fraction of cells without micronuclei were well positively correlated for acute gamma irradiation; no statistically significant positive correlation was obtained for chronic gamma irradiation. This indicates that the cellular mechanism that causes micronuclei formation is only partly responsible for the loss of reproductive integrity of irradiated cells. The trend is steep for chronic exposure, indicating that several inactivated cells are not micronucleated. This was also observed, to a lesser extent, for acute exposure.

### 4.2 Acute and chronic alpha particle exposures

The ratio of α-coefficients for micronucleus induction due to alpha exposures at high and low dose rates was close to 1, indicating that, at low doses, the MN yield was about the same for chronic and acute irradiation. The absence of greater efficacy for acute vs. chronic alpha exposure could be explained by considering the characteristics of exposure to alpha particles at the doses considered. For both acute and chronic alpha particle exposures, the inter-track contribution is expected to be small compared with the intra-track contribution. In fact, for example, at the highest dose considered, 0.9 Gy, the average number of alpha particles interacting inside the nucleus is small (equal to 7) with a total exposure duration of a few minutes for acute irradiation (at 4,980 mGy/h) and of 50 h for chronic irradiation (at 18 mGy/h). The lack of alpha tracks severely limits the chances that DSBs produced by different tracks would be close enough to interact. Probably, the inter-track contribution becomes significant only at higher doses than those considered in our experiment. Moreover, significant SLD is not expected for alpha particles. Thus, at low doses, the final DNA damage due to alpha particles could simply result from the sum of the uncorrected DNA repair induced by the individual alpha tracks during irradiation. This is in line with the results obtained considering the dose rate of 5 mGy/h; in fact, the mean number of micronuclei per BNC is the same at both 18 and 5 mGy/h, regardless of the average time interval between the arrival of one particle and the next (~7 and 23 h for 18 and 5 mGy/h, respectively, at the dose of 0.4Gy). However, surprisingly, chronic irradiation was more efficient at inducing micronucleated cells than acute irradiation at doses >0.1 Gy. This difference appears to increase as the dose and total exposure time increase. The ratio of the mean numbers of micronuclei per BNC per unit dose at the highest dose and dose rate considered (0.9 Gy, 4,980 mGy/h) to the mean numbers of micronuclei per BNC per unit dose at the lowest dose and dose rate considered (0.1 Gy, 18 mGy/h) was ~0.5. This inverse dose-rate effect was first obtained in confluent human fibroblasts for cytogenetic damage expressed as micronuclei induced by alpha particles and was the main new finding obtained in our study. Such an inverse dose-rate effect has also been observed in epidemiological studies of lung cancer for radon-exposed underground miners (69) and in studies of radon inhalation in rats (70). Moreover, several previous studies have shown an inverse dose-rate effect for neoplastic transformation using mammalian cells exposed to high-LET radiation (at dose rates below ~ 0.5 cGy/min, i.e., ~300 mGy/h). Hill et al. (71–73) showed that the incidence of transformation in C3H 10T1/2 mouse embryonic fibroblasts induced by a dose of 21 cGy of neutrons with a mean energy of 0.85 MeV was enhanced if the dose was given continuously at a low dose rate (below 0.43 cGy/min) or delivered in a series of dose fractions over 5 days compared with a single acute exposure. Miller et al. (74–76) also reported that fractionating low doses of neutrons or of deuterons and helium nuclei (with LET values between 40 and 120 keV/μm) results in enhanced transformation effects for C3H 10T1/2 cells. This finding is further supported by the results of Bettega et al. (77), who showed an enhanced transformation effect of fractionation in C3H 10T1/2 cells by 4.3 MeV alpha particles (LET = 101 keV/μm). In contrast, no inverse dose-rate effect for neoplastic transformation was found by Hieber et al. in C3H 10T1/2 with alpha particles of 2.7 MeV, LET = 147 keV/micron (78). All these studies found the inverse dose-rate effect for neoplastic transformation in cycling C3H 10T1/2 cells (cultures growing asynchronously). It seems to disappear for plateau-phase cells (74). A suggested mechanism to explain this effect has been to assume the presence of a period of high sensitivity to transformation during the cell cycle. This mechanism cannot also be applied to explain the inverse dose-rate effect observed for micronucleus induction since confluent cells were considered. We hypothesized two effects that could explain the greater effectiveness of chronic irradiation compared with acute irradiation in inducing micronuclei. The first is a cell cycle effect: a more pronounced delay effect could occur for acute irradiation than chronic irradiation. We found a significantly lower nuclear division index value for high-dose-rate exposure than for low-dose-rate exposure at 0.9 Gy, indicating more pronounced blocks of mitotic activities. A greater percentage of cells may miss the harvesting window in 72-h culture (or die due to interphase death) following acute irradiation compared to chronic irradiation. The second is the bystander effect as a manifestation of intercellular communication, which for chronic irradiation may increase as the dose increases, increasing the time interval in which the cells are in contact (5.3 h for 0.1 Gy, 21 h for 0.4 Gy and 48 h for 0.9 Gy). As is known, the “bystander effect” refers to the response of cells not directly irradiated but close to irradiated cells at the time of irradiation and receiving their signals either via gap junctions or through soluble factors. To obtain information on the occurrence of the bystander effect, only a fraction of the cells in the population must be irradiated. This can be achieved in several ways, i.e., with either a microbeam or a broad beam. We followed this second approach. Two methods can be used to perform bystander studies with a broad beam: partial shielding of the irradiated cell population or considering doses low enough to spare a meaningful fraction of the population cells. We chose this second method considering an average dose delivered to a cell population of 0.006 Gy (only for alpha exposure). For such low average dose values, a large dose heterogeneity within the cell population can be achieved; therefore, this situation represents the typical scenario for involvement in bystander effects. In the absence of cellular communication, the percentage of BNC with micronuclei in a cell population exposed to alpha particles is expected to be directly proportional to the fraction of cell nuclei hit by alpha particles (randomly distributed among the whole cell population). At the dose of 0.1 Gy, for which the percentage of hit nuclei is 55.7% (see Table 1), we found a net mean percentage of BNC with micronuclei of 7.8% in the case of chronic exposure. If the percentage of nuclei hit by alpha particles is reduced ~12-fold compared to the previous one (that is, to ~5%), ~12-fold reduction in the percentage of BNC with micronuclei should be obtained. Instead, the net mean percentage of BNC with micronuclei induced by the chronically delivered dose of 0.006 Gy, for which the percentage of hit nuclei by alpha particles is 4.8% (see Table 1), was of 1.1%, which is only seven times lower than that found at a dose of 0.1 Gy. Therefore, this indicates that in the cell population, there could also be cells not hit by alpha particles that, due to communication with the neighboring hit cells, undergo cytogenetic damage that originates in a micronucleus. However, this could also be due to a greater delay in cell proliferation for the 0.1 Gy dose than for the 0.006 Gy dose, in which case it would not be a bystander effect but a cell cycle delay effect. The equality of cytogenetic damage in terms of MN frequencies for chronic and acute exposures at the 0.1 Gy dose would suggest a greater contribution of the cell cycle effect than cell communication.

The supposedly greater efficacy of the chronic beam compared to the acute beam, indicated by the micronucleus data at doses ≥0.4 Gy, was not observed for cell survival. The effectiveness of inducing cell killing for chronic exposure to alpha particles was equal to that for acute exposure for all doses considered, indicating the absence of a one-to-one correlation between micronucleated cells and inactivated cells. This result was in agreement with data obtained by other authors. Yang et al. found the same survival curves by irradiating C3H 1OT1/2 cells in the plateau phase with 600 MeV/*n* iron ions (LET = 200 keV/μm) at a low (0.02 Gy/min) and high (1 Gy/min) dose rate. In addition, they found that there was no systematic increase or decrease in cell survival for a wide range of dose rates, from 0.016 to 1.5 Gy/min, by exposing confluent C3H 1OT1/2 cells to argon ions of 400 MeV/*n* (LET = 120 keV/μm) (79). Miller et al. obtained the same survival curves by irradiating exponentially growing C3H 1OT1/2 cells with single or fractional neutron exposures (74).

A positive correlation between the surviving fraction and the fraction of cells without micronuclei was also observed for alpha particle exposure. These results were in agreement with the data found by other authors for different types of cells and radiations (13, 40, 41). However, the data were fitted by different polynomial functions of the second degree for the high- and low-dose-rate irradiations and for the low and high LET irradiations. The observation of distinct relationships for each type of radiation considered suggests a dissociation (differentiation) of the mechanisms involved. Moreover, the fraction of micronucleated cells is significantly lower than that of inactivated cells, as obtained for gamma exposure. This indicates that the radiation damage that leads to the presence of micronuclei represents a subset of the lethal damage leading to the reproductive death of irradiated cells. The detection of the micronucleus frequency in BNC alone in AG1522 cells is insufficient to measure radiation-induced injury and to predict reproductive cell death in a general way. Other severe chromosomal aberrations (dicentrics plus rings plus large deletions, chromosomal breaks, nucleoplasmic bridges, etc.) should be considered, leading to apoptotic cell death or senescence before entry into mitosis or to mitotic catastrophe with an irreversible arrest of the cell cycle. Depending on the balance between certain proteins in the cell, mitotic catastrophe can end in a lethal process, such as apoptosis, autophagic cell death, or necrosis, or a cell survival program can be triggered by the development of cellular senescence (80).

The results may give insight into the mechanisms involved in chronic alpha particle exposure of confluent AG1522 cells. The observation that the damage induced by acute and chronic alpha exposure is the same at the 0.1 Gy dose suggests that the cellular response mechanisms might be the same for high and low dose rates at low doses. The greater cytogenetic damage observed for chronic vs. acute exposures at doses of 0.4 and 0.9 Gy is probably not linked to the mode of damage induction since the only significant contribution for doses below 1 Gy is the intra-track contribution for both chronic and acute exposures. This suggests that at doses ≥0.4 Gy, the cellular response mechanisms to the initial damage induced after chronic exposure may differ from those implemented after acute exposure. Probably for chronic exposure, these mechanisms are influenced by both the total time of exposure and the temporal distance between the traversing of the cell by one alpha particle and the next. At such doses (≥ 0.4 Gy), the total chronic exposure times, >20 h, are much greater than acute exposure times (on the order of minutes). At both high and low dose rates, the alpha particles hit the same stationary cells in the G0/G1 phase (~3 and 7 alpha particles per cell at doses of 0.4 Gy and 0.9 Gy, see Table 3) since the exposures were carried out on confluent cells. However, unlike acute exposure, where the average time interval between the traversing of the cell by one alpha particle and the next is a few minutes, that interval is ~7 h for chronic exposure. In the latter case, for the first 7 h of exposure, the cellular response mechanisms should only act on the damage induced by the first alpha particle. Furthermore, for chronic exposures at doses ≥0.4 Gy, the cellular communication mechanisms would have a very long time to act by promoting the exchange of signal molecules through gap junctions or soluble factors in the culture medium. The effect of such cellular communication would be in addition to the direct effect of radiation since all cells in the population are traversed by alpha particles (at doses ≥0.4 Gy). Although, in this case, the mechanisms involved after chronic low-dose exposure might differ from those involved after acute exposure, they lead to the same percentage of dead cells (see Figure 8). Further future studies will be needed to clarify this point and increase knowledge about the mechanisms involved.

### 4.3 Gamma-ray vs. alpha-particle exposures

RBE values for micronucleus induction can be calculated from the ratio of the α-coefficients for alpha particles and gamma rays. Values of ~5 and 7 for acute and chronic alpha exposures were obtained for RBE_ɤ*Acute*_. These values are in agreement with the results found by other authors. Paterson et al. examined the RBE of DNA damage generated by high LET secondary particles (protons and ^14^C nuclei) produced by thermal neutrons in human peripheral blood lymphocytes (81). They found that the RBE for micronucleus induction was higher at low doses, with a maximum value of 9, and decreased with increasing doses, revealing a minimum value of 7. Manti et al. (40) observed an RBE value of ~3 for the micronucleus induction by alpha particles in Chinese hamster V79 cells, considering levels of frequency of micronuclei in the range of 20%−40%. Considering chronic gamma rays as reference radiation, much larger RBE values for MN induction by alpha particles were found. RBE_γ*Chronic*_ values were ~19 and 25 for acute and chronic alpha exposure, respectively, obtaining an RBE_γ*Chronic*_/RBE_γ*Acute*_ ratio equal to ~4. However, these RBE values might be overestimated. The doubling time of AG1522 cells was 28 h, and the treatment time with cytochalasin-B was 72 h. Some damaged AG1522 cells may not be blocked by cytochalasin B during the 72 h and may not be counted. This may result in an underestimation of the level of MN induced, especially by gamma rays.

Similarly, different RBE values for cell killing by alpha particles were obtained from the ratio of the α-coefficients for alpha particles and gamma rays by taking low- or high-dose rate gamma rays as reference radiation. An RBE_S, γ*Acute*_ value equal to 4 was found, which was in agreement with other data obtained in the literature. An RBE_S, γ*Acute*_ value of 5 was obtained by Autsavapromporn et al., who considered AG1522 fibroblasts in the confluent state irradiated with gamma rays and alpha particles (43). Manti et al. (40) also found an RBE_S, γ*Acute*_ value of ~4, considering Chinese hamster V79 cells irradiated with alpha particles and 300 kVp X-rays. Finally, an RBE_S, γ*Acute*_ for cell killing by alpha particles equal to 3.9 and 5.0 for 10 and 50% survival, respectively, was determined by Raju et al. (37) considering AG1522 cells. The RBE value obtained by considering chronic gamma rays as the reference radiation was ~17 (much higher than that obtained by considering acute gamma rays as reference radiation due to the repair of sublethal damage). The RBE_S, γ*Chronic*_/RBE_S, γ*Acute*_ ratio was equal to 4.3.

## 5 Conclusion

In this study, a robust comparison of the biological effects induced by chronic and acute irradiation at low and high LET was made for the first time by considering the same *in vitro* biological system and experimental setup. This is of particular importance in the case of chronic exposures, where making comparisons using the results of different articles/studies in the literature could lead to inaccurate or even incorrect conclusions due to differences in factors other than radiation. In the case of chronic exposures at both low and high LET, a dose rate value of 18 mGy/h was chosen, for which few *in vitro* radiobiological data are available. *In vitro* cell measurements were also carried out at a dose rate of 5 mGy/h, corresponding to the definition of low dose rates given by UNSCEAR for radiations such as external X-rays and gamma rays (82).

The new findings obtained in our study concerned the effects (in terms of micronuclei and survival) induced by exposures at low dose rates (≤ 18 mGy/h) on confluent human fibroblasts, compared with those at high dose rates. For chronic gamma exposure, new information has been added, for example, regarding the SLDR, and other results were in agreement with those obtained by other authors using different cell lines or dose rates (13, 15, 23). To the best of our knowledge, no similar studies have been conducted to investigate the induction of micronuclei in confluent human fibroblasts by low- and high-dose-rate alpha particles. The main new finding obtained in our study concerned an inverse dose-rate effect observed for our cellular system exposed to alpha particles. This effect was similar to that found for neoplastic transformation in cycling C3H 1OT1/2 cells (76). In addition, other important results obtained from the comparison of low- and high-LET radiation dose rates are as follows:

1. Acute and chronic gamma-ray exposures comparison: chronic low-dose gamma exposures were 3.5 times and 4.1 times less effective than acute high-dose gamma exposures in producing micronucleated cells and dead cells in confluent human fibroblasts AG1522. The results obtained for cell survival would suggest a DDREF value for gamma-ray exposures of 8.8, considering cell killing as an indicator. However, this DDREF value cannot be automatically applied to humans. In fact, *in vitro* cell systems are much simpler model systems than humans, where cells are contained in tissues, forming organs with specific functions.
2. A one-to-one correlation was not observed between micronucleus induction and cell inactivation.

In conclusion, the results could provide important information for low-dose/dose rate modeling. Moreover, they may yield important insights applicable to all situations involving prolonged exposure to both low- and high-LET radiation, such as in earth and space radiation protection, nuclear medicine diagnostics and theragnostics, and biodosimetry.

## Data Availability

The original contributions presented in the study are included in the article/Supplementary material, further inquiries can be directed to the corresponding author.

## References

[1] Belli M Cherubini R Dalla Vecchia M Dini V Esposito G Moschini G et al. DNA fragmentation in V79 cells with light ions as measured by pulsed-field gel electrophoresis. I Experimental results. Int J Radiat Biol. (2002) 78:475–82. 10.1080/09553000210123676 12065052

[2] Pearson DD Provencher L Brownlee PM Goodarzi AA. Modern sources of environmental ionizing radiation exposure and associated health consequences. In:Kovalchuk I Kovalchuk O, editor. Genome Stability, 2nd Edn. Cambridge, MA: Academic Press (2021), p. 603–19. 10.1016/B978-0-323-85679-9.00032-5

[3] Penninckx S Pariset E Cekanaviciute E Costes SV. Quantification of radiation-induced DNA double-strand break repair foci to evaluate and predict biological responses to ionizing radiation. NAR Cancer. (2021) 3:zcab046. 10.1093/narcan/zcab046 35692378 PMC8693576

[4] Tang FR Loke WK Khoo BC. Low-dose or low-dose-rate ionizing radiation-induced bioeffects in animal models. J Radiat Res. (2017) 58:165–82. 10.1093/jrr/rrw120 28077626 PMC5439383

[5] Paunesku T Stevanović A Popović J Woloschak GE. Effects of low dose and low dose rate low linear energy transfer radiation on animals - review of recent studies relevant for carcinogenesis. Int J Radiat Biol. (2021) 97:757–68. 10.1080/09553002.2020.1859155 33289582 PMC9216178

[6] Elbakrawy EM Mayah A Hill MA Kadhim M. Induction of genomic instability in a primary human fibroblast cell line following low-dose alpha-particle exposure and the potential role of exosomes. Biology. (2021) 10:11. 10.3390/biology10010011 33379152 PMC7824692

[7] Belli M Indovina L. The response of living organisms to low radiation environment and its implications in radiation protection. Front Public Health. (2020) 8:601711. 10.3389/fpubh.2020.601711 33384980 PMC7770185

[8] Dobney W Mols L Mistry D Tabury K Baselet B Baatout S. Evaluation of deep space exploration risks and mitigations against radiation and microgravity. Front Nucl Med. (2023) 3:1225034. 10.3389/fnume.2023.1225034 39355042 PMC11440958

[9] International Atomic Energy Agency (IAEA). Patient Radiation Exposure Monitoring in Medical Imaging. Safety Reports Series No. 112. Vienna: IAEA (2023).

[10] Barquinero JF Fattibene P Chumak V Ohba T Della Monaca S Nuccetelli C et al. Lessons from past radiation accidents: critical review of methods addressed to individual dose assessment of potentially exposed people and integration with medical assessment. Environ Int. (2021) 146:106175. 10.1016/j.envint.2020.106175 33069983

[11] Danforth JM Provencher L Goodarzi AA. Chromatin and the cellular response to particle radiation-induced oxidative and clustered DNA damage. Front Cell Dev Biol. (2022) 13:10:910440. 10.3389/fcell.2022.910440 35912116 PMC9326100

[12] Hada M Georgakilas AG. Formation of clustered DNA damage after high-LET irradiation: a review. J Radiat Res. (2008) 49:203–10. 10.1269/jrr.07123 18413977

[13] Geard CR Chen CY. Micronuclei and clonogenicity following low- and high-dose-rate gamma irradiation of normal human fibroblasts. Radiat Res. (1990) 124:S56–61. 10.2307/3577678 2236512

[14] Bhat NN Rao BS. Dose rate effect on micronuclei induction in cytokinesis blocked human peripheral blood lymphocytes. Radiat Prot Dosimetry. (2003) 106:45–52. 10.1093/oxfordjournals.rpd.a006333 14653325

[15] Nakamura H Fukami H Hayashi Y Tachibana A Nakatsugawa S Hamaguchi M et al. Cytotoxic and mutagenic effects of chronic low-dose-rate irradiation on TERT-immortalized human cells. Radiat Res. (2005) 163:283–8. 10.1667/RR3310 15733035

[16] Little MP Kitahara CM Cahoon EK Bernier MO Velazquez-Kronen R Doody MM et al. Occupational radiation exposure and risk of cataract incidence in a cohort of US radiologic technologists. Eur J Epidemiol. (2018) 33:1179–91. 10.1007/s10654-018-0435-3 30151727 PMC10645574

[17] Barnard SGR McCarron R Moquet J Quinlan R Ainsbury E. Inverse dose-rate effect of ionising radiation on residual 53BP1 foci in the eye lens. Sci Rep. (2019) 9:10418. 10.1038/s41598-019-46893-3 31320710 PMC6639373

[18] Barnard SGR McCarron R Mancuso M De Stefano I Pazzaglia S Pawliczek D et al. Radiation-induced DNA damage and repair in lens epithelial cells of both Ptch1(+/-) and Ercc2(+/-) mutated mice. Radiat Res. (2022) 197:36–42. 10.1667/RADE-20-00264.1 33652474

[19] Campa A Balduzzi M Dini V Esposito G Tabocchini MA. The complex interactions between radiation induced non-targeted effects and cancer. Cancer Lett. (2015) 356:126–36. 10.1016/j.canlet.2013.09.030 24139968

[20] Morgan WF. Non-targeted and delayed effects of exposure to ionizing radiation: I. Radiation-induced genomic instability and bystander effects *in vitro*. Radiat Res. (2003) 159:567–80. 10.1667/0033-7587(2003)159[0567:NADEOE]2.0.CO;2 12710868

[21] Kanagaraj K Rajan V Pandey BN Thayalan K Venkatachalam P. Primary and secondary bystander effect and genomic instability in cells exposed to high and low linear energy transfer radiations. Int J Radiat Biol. (2019) 95:1648–58. 10.1080/09553002.2019.1665208 31486717

[22] Tang H Cai L He X Niu Z Huang H Hu W et al. Radiation-induced bystander effect and its clinical implications. Front Oncol. (2023) 13:1124412. 10.3389/fonc.2023.1124412 37091174 PMC10113613

[23] de Toledo SM Asaad N Venkatachalam P Li L Howell RW Spitz DR et al. Adaptive responses to low-dose/low-dose-rate gamma rays in normal human fibroblasts: the role of growth architecture and oxidative metabolism. Radiat Res. (2006) 166:849–57. 10.1667/RR0640.1 17149977

[24] Dainiak N Feinendegen LE Hyer RN Locke PA Waltar AE. Synergies resulting from a systems biology approach: integrating radiation epidemiology and radiobiology to optimize protection of the public after exposure to low doses of ionizing radiation. Int J Radiat Biol. (2018) 94:2–7. 10.1080/09553002.2018.1407461 29172847

[25] Akuwudike P López-Riego M Marczyk M Kocibalova Z Brückner F Polańska J et al. Short- and long-term effects of radiation exposure at low dose and low dose rate in normal human VH10 fibroblasts. Front Public Health. (2023) 11:1297942. 10.3389/fpubh.2023.1297942 38162630 PMC10755029

[26] Brooks AL Eberlein PE Couch LA Boecker BB. The role of dose-rate on risk from internally-deposited radionuclides and the potential need to separate the dose-rate effectiveness factor (DREF) from the dose and dose-rate effectiveness factor (DDREF). Health Phys. (2009) 97:458–69. 10.1097/HP.0b013e3181ac910e 19820455

[27] Lowe D Roy L Tabocchini MA Rühm W Wakeford R Woloschak GE. Radiation dose rate effects: what is new and what is needed? Radiat Environ Biophys. (2022) 61:507–43. 10.1007/s00411-022-00996-0 36241855 PMC9630203

[28] Tang FR Loganovsky K. Low dose or low dose rate ionizing radiation-induced health effect in the human. J Environ Radioact. (2018) 192:32–47. 10.1016/j.jenvrad.2018.05.018 29883875

[29] Sørensen BS Overgaard J Bassler N. *In vitro* RBE-LET dependence for multiple particle types. Acta Oncol. (2011) 50:757–62. 10.3109/0284186X.2011.582518 21767171

[30] Esposito G Belli M Simone G Sorrentino E Tabocchini MA. A 244Cm irradiator for protracted exposure of cultured mammalian cells with alpha particles. Health Phys. (2006) 90:66–73. 10.1097/01.HP.0000175167.61317.cc 16340609

[31] Esposito G Antonelli F Belli M Campa A Simone G Sorrentino E et al. An alpha-particle irradiator for radiobiological research and its implementation for bystander effect studies. Radiat Res. (2009) 172:632–42. 10.1667/RR1697.1 19883232

[32] Esposito G Anello P Pecchia I Tabocchini MA Campa A. Facility for gamma irradiations of cultured cells at low dose rates: design, physical characteristics and functioning. Appl Radiat Isot. (2016) 115:227–34. 10.1016/j.apradiso.2016.06.018 27423023

[33] ICRP. Relative biological effectiveness (RBE), quality factor (Q), and radiation weighting factor (w(R)). A report of the International Commission on Radiological Protection. Ann ICRP. (2003) 33:1–117. 10.1016/S0146-6453(03)00024-1 14614921

[34] Fenech M Morley AA. Measurement of micronuclei in lymphocytes. Mutat Res. (1985) 147:29–36. 10.1016/0165-1161(85)90015-9 3974610

[35] Fenech M Chang WP Kirsch-Volders M Holland N Bonassi S Zeiger E. project: detailed description of the scoring criteria for the cytokinesis-block micronucleus assay using isolated human lymphocyte cultures. Mutat Res. (2003) 534:65–75. 10.1016/S1383-5718(02)00249-8 12504755

[36] Eastmond DA Tucker JD. Identification of aneuploidy-inducing agents using cytokinesis-blocked human lymphocytes and an antikinetochore antibody. Environ Mol Mutagen. (1989) 13:34–43. 10.1002/em.2850130104 2783409

[37] Raju MR Eisen Y Carpenter S Inkret WC. Radiobiology of alpha particles. III Cell inactivation by alpha-particle traversals of the cell nucleus. Radiat Res. (1991) 128:204–9. 10.2307/3578139 1947017

[38] Azzam EI de Toledo SM Gooding T Little JB. Intercellular communication is involved in the bystander regulation of gene expression in human cells exposed to very low fluences of alpha particles. Radiat Res. (1998) 150:497–504. 10.2307/3579865 9806590

[39] Bettega D Calzolari P Doneda L Durante M Tallone L. Early and delayed reproductive death in human cells exposed to high energy iron ion beams. Adv Space Res. (2005) 35:280–5. 10.1016/j.asr.2005.01.060 15934207

[40] Manti L Jamali M Prise KM Michael BD Trott KR. Genomic instability in Chinese hamster cells after exposure to X rays or alpha particles of different mean linear energy transfer. Radiat Res. (1997) 147:22–8. 10.2307/3579438 8989365

[41] Sgura A Antoccia A Cherubini R Dalla Vecchia M Tiveron P Degrassi F et al. Micronuclei, CREST-positive micronuclei and cell inactivation induced in Chinese hamster cells by radiation with different quality. Int J Radiat Biol. (2000) 76:367–74. 10.1080/095530000138709 10757316

[42] Raju MR Eisen Y Carpenter S Jarrett K Harvey WF. Radiobiology of alpha particles. IV Cell inactivation by alpha particles of energies 04-35 MeV. Radiat Res. (1993) 133:289–96. 10.2307/3578212 8451379

[43] Autsavapromporn N de Toledo SM Little JB Gerin J-PJ Harris AL Azzam EI. The role of gap junction communication and oxidative stress in the propagation of toxic effects among high-dose α-particle-irradiated human cells. Radiat Res. (2011) 175:347–57. 10.1667/RR2372.1 21388278 PMC3139025

[44] Franken NAP. ten Cate R Krawczyk PM Stap J Haveman J Aten J. Comparison of RBE values of high-LET α-particles for the induction of DNA-DSBs, chromosome aberrations and cell reproductive death. Radiat Oncol. (2011) 6:64. 10.1186/1748-717X-6-64 21651780 PMC3127784

[45] Guerra Liberal FDC Thompson SJ Prise KM McMahon SJ. High-LET radiation induces large amounts of rapidly-repaired sublethal damage. Sci Rep. (2023) 13:11198. 10.1038/s41598-023-38295-3 37433844 PMC10336062

[46] Gersey B Sodolak J Hada M Saganti P Wilkins R Cucinotta F et al. Micronuclei induction in human fibroblasts exposed *in vitro* to Los Alamos high-energy neutrons. Adv Space Res. (2007) 40:1754–7. 10.1016/j.asr.2007.03.018

[47] George KA Hada M Cucinotta FA. Biological effectiveness of accelerated protons for chromosome exchanges. Front Oncol. (2015) 5:226. 10.3389/fonc.2015.00226 26539409 PMC4610205

[48] Kaspler P Chen R Hyrien O Jelveh S Bristow RG Hill RP. Biodosimetry using radiation-induced micronuclei in skin fibroblasts. Int J Radiat Biol. (2011) 87:824–38. 10.3109/09553002.2011.582927 21801108

[49] Qutob SS Ng CE. Comparison of apoptotic, necrotic and clonogenic cell death and inhibition of cell growth following camptothecin and X-radiation treatment in a human melanoma and a human fibroblast cell line. Cancer Chemother Pharmacol. (2002) 49:167–75. 10.1007/s00280-001-0403-5 11862432

[50] Słonina D Spekl K Panteleeva A Brankovic K Hoinkis C Dörr W. Induction of micronuclei in human fibroblasts and keratinocytes by 25 kV X-rays. Radiat Environ Biophys. (2003) 42:55–61. 10.1007/s00411-003-0177-8 12720002

[51] Litvinchuk AV Vachelová J Michaelidesová A Wagner R Davídková M. Dose-dependent micronuclei formation in normal human fibroblasts exposed to proton radiation. Radiat Environ Biophys. (2015) 54:327–34. 10.1007/s00411-015-0598-1 25972267

[52] Cornforth M Shuryak I Loucas B. Lethal and nonlethal chromosome aberrations by gamma rays and heavy ions: a cytogenetic perspective on dose fractionation in hadron radiotherapy. Transl Cancer Res. (2017) 6:S769–78. 10.21037/tcr.2017.05.16

[53] Goodhead DT. Spatial and temporal distribution of energy. Health Phys. (1988) 55:231–40. 10.1097/00004032-198808000-00015 3410690

[54] Nikjoo H Uehara S Wilson WE Hoshi M Goodhead DT. Track structure in radiation biology: theory and applications. Int J Radiat Biol. (1998) 73:355–64. 10.1080/095530098142176 9587072

[55] Goodhead DT. Mechanisms for the biological effectiveness of high-LET radiations. J Radiat Res. (1999) 40(Suppl):1–13. 10.1269/jrr.40.S1 10804988

[56] Shin W-G Sakata D Lampe N Belov O Tran NH Petrovic I et al. A Geant4-DNA evaluation of radiation-induced DNA damage on a human fibroblast. Cancers. (2021) 13:4940. 10.3390/cancers13194940 34638425 PMC8508455

[57] Hsiao Y-Y Chen F-H Chan C-C Tsai C-C. Monte Carlo simulation of double-strand break induction and conversion after ultrasoft X-rays irradiation. Int J Mol Sci. (2021) 22:11713. 10.3390/ijms222111713 34769142 PMC8583805

[58] Tsai J-Y Chen F-H Hsieh T-Y Hsiao Y-Y. Effects of indirect actions and oxygen on relative biological effectiveness: estimate of DSB induction and conversion induced by gamma rays and helium ions. J Radiat Res. (2015) 56:691–9. 10.1093/jrr/rrv025 25902742 PMC4497398

[59] Nikjoo H O'Neill P Terrissol M Goodhead DT. Quantitative modelling of DNA damage using Monte Carlo track structure method. Radiat Environ Biophys. (1999) 38:31–8. 10.1007/s004110050135 10384953

[60] Stenerlöw B Höglund E Carlsson J Blomquist E. Rejoining of DNA fragments produced by radiations of different linear energy transfer. Int J Radiat Biol. (2000) 76:549–57. 10.1080/095530000138565 10815636

[61] Antonelli F Belli M Cuttone G Dini V Esposito G Simone G et al. Induction and repair of DNA double-strand breaks in human cells: dephosphorylation of histone H2AX and its inhibition by calyculin A. Radiat Res. (2005) 164:514–7. 10.1667/RR3379.1 16187759

[62] Hill MA. Radiation track structure: how the spatial distribution of energy deposition drives biological response. Clin Oncol. (2020) 32:75–83. 10.1016/j.clon.2019.08.006 31511190

[63] Schmid E Bauchinger M Mergenthaler W. Analysis of the time relationship for the interaction of X-ray induced primary breaks in the formation of dicentric chromosomes. Int J Radiat Biol Relat Stud Phys Chem Med. (1976) 30:339–46. 10.1080/09553007614551111 1086845

[64] Elbakrawy EM Hill MA Kadhim MA. Radiation-induced chromosome instability: the role of dose and dose rate. Genome Integr. (2019) 10:3. 10.4103/genint.genint_5_19 31897286 PMC6862263

[65] Hintzsche H Hemmann U Poth A Utesch D Lott J Stopper H. Fate of micronuclei and micronucleated cells. Mutat Res Rev Mutat Res. (2017) 771:85–98. 10.1016/j.mrrev.2017.02.002 28342454

[66] Reimann H Stopper H Hintzsche H. Fate of micronuclei and micronucleated cells after treatment of HeLa cells with different genotoxic agents. Arch Toxicol. (2023) 97:875–89. 10.1007/s00204-022-03433-9 36564592 PMC9968706

[67] Hall EJ Giaccia AJ. Radiobiology for the Radiologist. Philadelphia, PA: Lippincott Williams &Wilkins (LWW) (2018).

[68] Liu M Lee S Liu B Wang H Dong L Wang Y. Ku-dependent non-homologous end-joining as the major pathway contributes to sublethal damage repair in mammalian cells. Int J Radiat Biol. (2015) 91:867–71. 10.3109/09553002.2015.1075178 26189733 PMC4748373

[69] Lubin JH Boice Jr JD Edling C Hornung RW Howe G Kunz E et al. Radon-exposed underground miners and inverse dose-rate (protraction enhancement) effects. Health Phys. (1995) 69:494–500. 10.1097/00004032-199510000-00007 7558839

[70] Monchaux G. Risk of fatal versus incidental lung cancer in radon-exposed rats: a reanalysis of French data. Arch Oncol. (2004) 12:7–12. 10.2298/AOO0401007M

[71] Hill CK Han A Elkind MM. Fission-spectrum neutrons at a low dose rate enhance neoplastic transformation in the linear, low dose region (0-10 cGy). Int J Radiat Biol Relat Stud Phys Chem Med. (1984) 46:11–5. 10.1080/09553008414551011 6611317

[72] Hill CK Carnes BA Han A Elkind MM. Neoplastic transformation is enhanced by multiple low doses of fission-spectrum neutrons. Radiat Res. (1985) 102:404–10. 10.2307/3576716 4070554

[73] Hill CK Zhu L. Energy and dose-rate dependence of neoplastic transformation and mutations induced in mammalian cells by fast neutrons. Radiat Res. (1991) 128:S53–9. 10.2307/3578002 1924749

[74] Miller RC Brenner DJ Geard CR Komatsu K Marino SA Hall EJ. Oncogenic transformation by fractionated doses of neutrons. Radiat Res. (1988) 114:589–98. 10.2307/3577129 3375445

[75] Miller RC Brenner DJ Randers-Pehrson G Marino SA Hall EJ. The effects of the temporal distribution of dose on oncogenic transformation by neutrons and charged particles of intermediate LET. Radiat Res. (1990) 124:S62–8. 10.2307/3577679 2236513

[76] Miller RC Randers-Pehrson G Hieber L Marino SA Richards M Hall EJ. The inverse dose-rate effect for oncogenic transformation by charged particles is dependent on linear energy transfer. Radiat Res. (1993) 133:360–4. 10.2307/3578222 8451387

[77] Bettega D Calzolari P Chiorda GN Tallone-Lombardi L. Transformation of C3H 10T1/2 cells with 43 MeV alpha particles at low doses: effects of single and fractionated doses. Radiat Res. (1992) 131:66–71. 10.2307/3578318 1626050

[78] Hieber L Ponsel G Roos H Fenn S Fromke E Kellerer AM. Absence of a dose-rate effect in the transformation of C3H 10T1/2 cells by alpha-particles. Int J Radiat Biol Relat Stud Phys Chem Med. (1987) 52:859–69. 10.1080/09553008714552451 3500927

[79] Yang TC-H Craise LM Mei M-T Tobias CA. Dose protraction studies with low- and high-LET radiations on neoplastic cell transformation *in vitro*. Adv Space Res. (1986) 6:137–47. 10.1016/0273-1177(86)90286-3 11537213

[80] Sazonova EV Petrichuk SV Kopeina GS Zhivotovsky B. A link between mitotic defects and mitotic catastrophe: detection and cell fate. Biol Direct. (2021) 16:25. 10.1186/s13062-021-00313-7 34886882 PMC8656038

[81] Paterson LC Festarini A Stuart M Ali F Costello C Boyer C et al. High-accuracy relative biological effectiveness values following low-dose thermal neutron exposures support bimodal quality factor response with neutron energy. Int J Mol Sci. (2022) 23:878. 10.3390/ijms23020878 35055062 PMC8779315

[82] UNSCEAR. UNSCEAR 2012 Report to the General Assembly, with Scientific Annexes. Sources, Effects and Risks of Ionizing Radiation. New York, NY: United Nations (2015).

