# Supplementary material for: Biological effects in normal human fibroblasts following chronic and acute irradiation with both low- and high-LET radiation
Source: Front Public Health. 2024 Oct 22;12:1404748. doi: 10.3389/fpubh.2024.1404748 (PMC11534685; doi:10.3389/fpubh.2024.1404748)
Supplement: Supplementary file 1 [file Data_Sheet_1.PDF]

Supplementary Table 1. Distributions of micronuclei by acute gamma irradiation on days 1, 7 and 12 of confluence at doses of 0.9 and 1.4 Gy. N = numbers of BN cells scored, X = numbers of MN observed, y = micronucleus frequency per BNC, SE = standard error. Controls were considered at day 1 and day 12 of confluence.

Dose (Gy)	Confluence days	N	X	MN distribution					y ± SE
				MN_0	MN_1	MN_2	MN_3	MN_4	
0	1	1114	39	1080	30	3	1		0.035 ± 0.006
0	12	986	38	953	28	5			0.039 ± 0.007
0.9	1	1038	132	922	104	9	2	1	0.127 ± 0.012
0.9	7	996	126	878	111	6	1		0.127 ± 0.011
1.4	1	1075	201	892	168	13	1	1	0.187 ± 0.013
1.4	12	997	207	818	155	21	2	1	0.208 ± 0.015
